# The metabolic domestication syndrome of budding yeast

**DOI:** 10.1073/pnas.2313354121

**Published:** 2024-03-08

**Authors:** Roland Tengölics, Balázs Szappanos, Michael Mülleder, Dorottya Kalapis, Gábor Grézal, Csilla Sajben, Federica Agostini, João Benhur Mokochinski, Balázs Bálint, László G. Nagy, Markus Ralser, Balázs Papp

**Affiliations:** ^a^Hungarian Centre of Excellence for Molecular Medicine - Biological Research Centre Metabolic Systems Biology Lab, Szeged 6726, Hungary; ^b^Synthetic and System Biology Unit, National Laboratory of Biotechnology, Institute of Biochemistry, Biological Research Centre, Hungarian Research Network, Szeged 6726, Hungary; ^c^Metabolomics Lab, Core facilities, Biological Research Centre, Hungarian Research Network, Szeged 6726, Hungary; ^d^Department of Biotechnology, University of Szeged, Szeged 6726, Hungary; ^e^Charité Universitätsmedizin, Core Facility High-Throughput Mass Spectrometry, Berlin 10117, Germany; ^f^Department of Biochemistry, Charité Universitätsmedizin, Berlin 10117, Germany; ^g^Institute of Biochemistry, Biological Research Centre, Hungarian Research Network, Szeged 6726, Hungary; ^h^The Francis Crick Institute, Molecular Biology of Metabolism Laboratory, London NW11AT, United Kingdom; ^i^National Laboratory for Health Security, Biological Research Centre, Hungarian Research Network, Szeged 6726, Hungary

**Keywords:** *Saccharomyces cerevisiae*, phylogenetic comparative methods, domestication, metabolomics, convergent evolution

## Abstract

Cellular metabolism evolves through changes in the structure and quantitative states of metabolic networks. Here, we explore the evolutionary dynamics of metabolic states by focusing on the collection of metabolite levels, the metabolome, which captures key aspects of cellular physiology. Using a phylogenetic framework, we profiled metabolites in 27 populations of nine budding yeast species, providing a graduated view of metabolic variation across multiple evolutionary time scales. Metabolite levels evolve more rapidly and independently of changes in the metabolic network’s structure, providing complementary information to enzyme repertoire. Although metabolome variation accumulates mainly gradually over time, it is profoundly affected by domestication. We found pervasive signatures of convergent evolution in the metabolomes of independently domesticated clades of *Saccharomyces cerevisiae*. Such recurring metabolite differences between wild and domesticated populations affect a substantial part of the metabolome, including rewiring of the TCA cycle and several amino acids that influence aroma production, likely reflecting adaptation to human niches. Overall, our work reveals previously unrecognized diversity in central metabolism and the pervasive influence of human-driven selection on metabolite levels in yeasts.

## Significance

Metabolic networks evolve through changes in enzyme content and activity states, but the latter aspect remains elusive. This is a major knowledge gap since differences in enzyme kinetic properties and regulation can lead to vastly different metabolic activities despite similar enzyme contents. Here, we profiled metabolite levels across several budding yeast species and populations to delineate the evolutionary dynamics of metabolic states at exceptional phylogenetic resolution. The approach allowed us to uncover a global signature of domestication that evolved convergently in independently domesticated groups of *Saccharomyces cerevisiae* and likely reflects adaptation to human-made niches. More broadly, our results show that studying metabolic evolution through metabolomics provides largely complementary information that cannot be obtained by studying enzyme content alone.

Metabolic networks are central to life, as they provide building blocks and energy for all cellular processes. While their fundamental tasks are universal, metabolic networks display remarkable evolutionary diversity (1–3). Such variation exists at two levels: in the composition of biochemical reactions, i.e., network structure, and in the functional states of metabolic networks, i.e., intracellular metabolite concentrations and reaction rates (fluxes). Although important insights have been gained by comparing the structure and production capability of metabolic networks among species (4–9), evolutionary diversity at the level of functional states is much less studied. This represents an important gap in our knowledge, as evolutionary changes in the kinetic properties and regulation of enzymes can yield wide variations in metabolic activities even between organisms with highly similar metabolic network structures. For example, altered allosteric regulation of a catabolic enzyme, instead of modification of pathway structure, played a key role in adaptation to a suboptimal nutrient source in *E. coli* during laboratory evolution (10).

The intracellular levels of metabolites are major determinants of metabolic fluxes (11). Consequently, the collection of metabolite levels, the metabolome, captures an important aspect of the functional state of metabolic networks (12). Recent advances in metabolomics techniques have allowed measurements of metabolite levels at scale (13, 14), opening the way to systematically characterize metabolome variation across evolutionary lineages. In fact, metabolome comparisons revealed substantial variation in metabolite levels between and within species (2, 3, 15–17). Such variation may directly contribute to important phenotypes, such as stress tolerance and flavor in yeasts (18, 19), agriculturally important traits in plants (15, 17) or longevity in mammals (2), and are likely shaped by adaptive evolution. In contrast, some variations in metabolite levels are likely non-functional (neutral), as evidenced by mutations that strongly impact metabolite levels without measurably altering fitness (20, 21). Indeed, it has been recently proposed that much of the between-species variation in tissue metabolite levels in mammals are selectively neutral which are allowed, rather than favored by natural selection (22). However, the evolutionary driving forces of metabolite levels remain largely unresolved due to a shortage of comparative studies, especially in microbes.

The unicellular budding yeast *Saccharomyces cerevisiae* is a widely used model organism for functional genomics and systems biology. Recently, it has also emerged as an important model for population genomics due to its worldwide distribution across a range of environments, including human-associated and wild niches. Whole genome sequences for >1,000 *S. cerevisiae* strains revealed a high degree of genetic diversity and several genetically distinct lineages (populations) in this species (23). Importantly, *S. cerevisiae* is the dominant species in the fermentation of various beverages and foods and has been domesticated on several independent occasions (19, 24). Domestication had a large impact on the sexual life cycle, stress tolerance, and fermentative growth capacity of this species, raising the possibility that it also dramatically altered the functional states of its metabolic network. However, the diversity of metabolite levels is poorly characterized in yeasts due to limited metabolite coverage and narrow representation of natural variation in earlier studies (3, 25). Thus, beyond a few specific metabolic traits (19, 26), it remains unknown how evolutionary adaptation to human-made environments (domestication) influenced the yeast metabolome.

Here, we studied variation in metabolite levels within and among species by focusing on a diverse strain set representing nine species of the *Saccharomycetaceae* family and 17 genetically distinct populations of *S. cerevisiae*. We employed multiple complementary metabolomic platforms to obtain a global picture of variation in central metabolites. Analysis of the data in a phylogenetic context allowed us to address several open questions. First, we examined the relationship between two major aspects of metabolic evolution across multiple timescales: divergence in metabolite levels and divergence in the metabolic network’s capacity to produce metabolites. Second, we examined the impact of domestication on the metabolome and tested whether independent domestication events converged on a similar metabolome signature. We found that metabolite levels evolve much more rapidly and largely independently of the network’s structural properties and display a global recurrent signature of adaptation to human-made niches.

## Results

### Metabolomics Reveals Substantial Metabolic Diversity in Budding Yeasts.

**Fig. 1.**
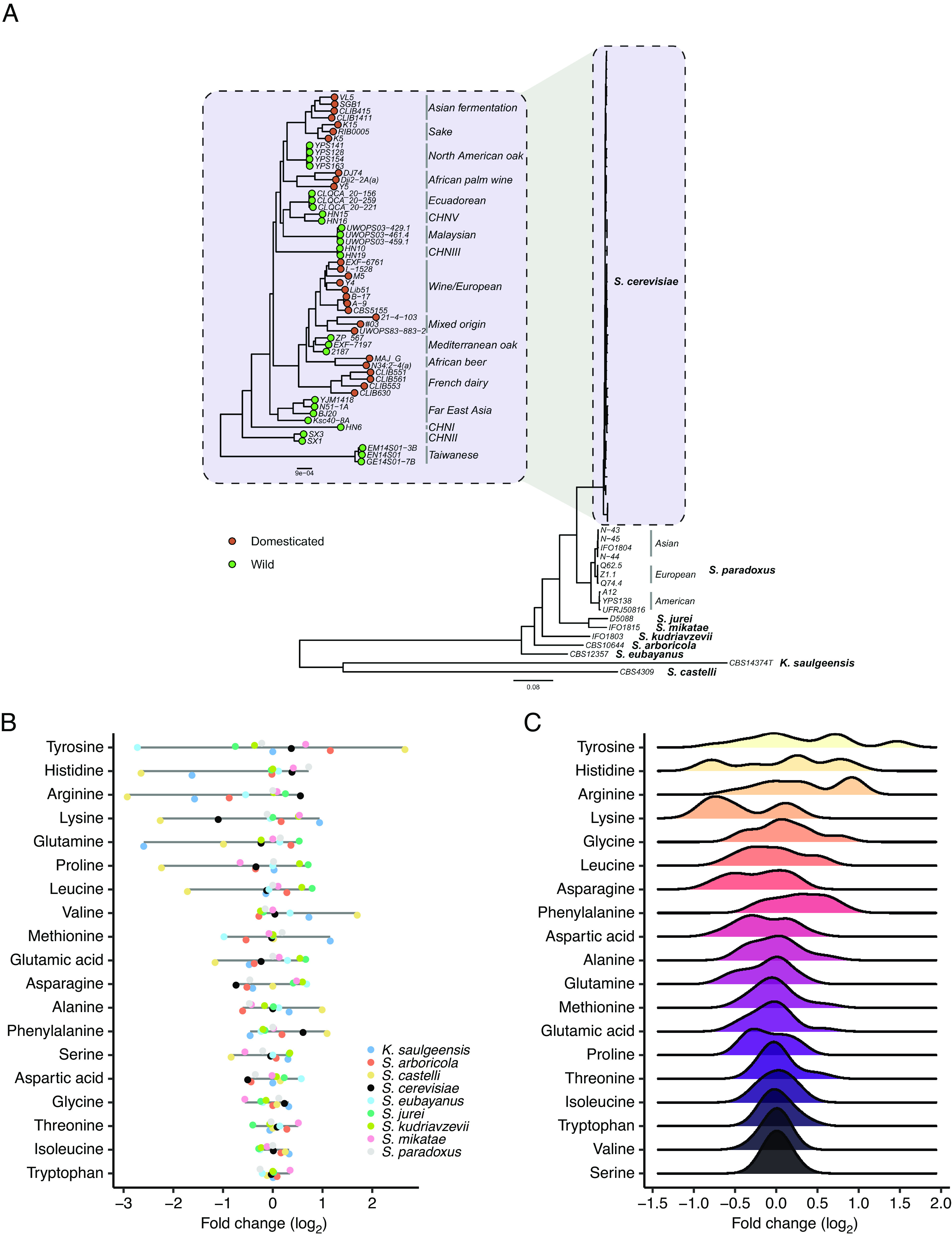
Phylogenetic diversity of amino acid levels in yeasts. (*A*) Maximum likelihood phylogenetic tree of yeast strains based on the alignment of 983 concatenated single-copy orthologs. Branch lengths represent the average number of substitutions per site. Species are indicated in bold, while strains and populations are indicated in italics. Green and orange dots denote wild and domesticated *S. cerevisiae* strains, respectively. Strains were considered as domesticated if the entire population is considered domesticated. (*B*) Metabolite fold change values for nine yeast species for each amino acid. Amino acids are ranked based on their between-species variance of fold change. Species-level fold change values were calculated by using species averages and using the median value of the species averages as a reference point. Colors represent species. (*C*) Distributions of metabolite fold change values across 17 *S. cerevisiae* populations, averaged by populations, for each amino acid. Amino acids are ranked based on their between-population variance of fold change. Population-level fold change values were calculated by using population averages and using the median value of the population averages as a reference point.

To study the evolutionary divergence of metabolomes, we focused on 71 yeast strains representing 27 populations of 9 budding yeast species and spanning ~90 My of evolution (Fig. 1 and Dataset S6). The set of isolates cover 17 genetically isolated populations of *S. cerevisiae*, including both wild and domesticated ones, and capture well the genetic and phenotypic differentiation of this species. Importantly, we only analyzed non-mosaic (clean) *S. cerevisiae* isolates to allow within-species inference of evolutionary history. Multiple isolates per population were included for *S. cerevisiae* and *Saccharomyces paradoxus*, where population structure has been well characterized. Even though some domesticated *S. cerevisiae* populations contain wild isolates as well (e.g., Asian Fermentation), we took care to represent such populations with only isolates collected from human-associated environments. Overall, the strain set studied here provides a graduated view of metabolome variation across multiple evolutionary time scales. Phylogenomic analysis of the strain set resulted in a phylogenetic tree that agrees well with previous studies (23, 27–30) and supports the basal position of wild isolates collected in Taiwan and China within *S. cerevisiae* (Fig. 1*A*, see *Methods* for details of the phylogenetic reconstruction).

We first measured the levels of 19 amino acids across the ~70 strains using a quantitative targeted metabolomics workflow. We primarily focused on amino acids as these are the building blocks of proteins and key intermediates of several biosynthetic pathways and therefore capture the activity of various metabolic processes (13). We collected metabolome data for all strains in the same defined growth environment and same growth phase (exponential) to minimize environmental variation and to reveal evolutionary differences in metabolite concentrations [i.e., common garden design (31)]. We used a synthetic minimal medium that is especially well suited to capture variation in biosynthetic metabolism (13) and a growth temperature (25 °C) that diminishes growth rate heterogeneity across strains (*SI Appendix*, Fig. S1). To exclude the possibility that metabolome differences are dominated by remaining growth differences among genotypes, we measured optical density (OD) of each culture at the time of sampling and applied a normalization strategy to remove potential OD-dependencies of metabolite levels (32, 33) (Dataset S1 and *Methods*).

We next characterized the extent of variation of amino acid levels across yeast species and populations by calculating the average values for each population (Fig. 1 *B* and *C*). We found substantial divergence in amino acid levels, with a 6.6-fold range among the different species when averaged across amino acids. As might be expected, the two phylogenetically most distantly related budding yeast species, *Kazachstania saulgeensis* and *Saccharomyces castelli*, show the largest deviations in several amino acids from the rest of the species (Fig. 1*B*). Statistical analysis confirmed that 18 out of 19 amino acids show significant variation across the 27 populations studied (ANOVA tests, *SI Appendix*, Table S1). Importantly, a similar conclusion holds even when comparing distinct populations of the same species, *S. cerevisiae* (17 out of 19 amino acids vary significantly, ANOVA tests, see *SI Appendix*, Table S1).

The degree of between-species divergence varies significantly among different amino acids (Bartlett’s test of homogeneity of variances was applied to the fold change values, *P* < 2.2e-16, Fig. 1*B*). Furthermore, amino acids also differ in their degree of divergence when measured among *S. cerevisiae* populations (Bartlett’s test, *P* < 2.2e-16, Fig. 1*C*). Ranking of amino acids based on their evolutionary variability suggests that the levels of tyrosine, histidine, arginine, and lysine diverged more extensively than those of tryptophan, isoleucine, and threonine, which appear more conserved (Fig. 1 *B* and *C*). Notably, both the between-species and the within-*S. cerevisiae* comparisons reveal this trend, indicating shared evolutionary forces shaping the metabolome across multiple phylogenetic time scales.

To estimate the phylogenetic diversity of metabolite levels beyond amino acids, we also measured 78 metabolites of primary metabolism, excluding amino acids, using a non-targeted metabolomics platform (Dataset S2 and *Methods*). Consistent with the amino acid data, we find that the majority of detected metabolites show significant variation between the 27 populations (74 out of 78, see Dataset S7). Together, these patterns reveal a previously hidden diversity of metabolic activities associated with central metabolites.

### Decoupled Evolution of Metabolite Levels and Metabolic Network Structure.

We next interrogated the relationship between metabolic evolution occurring at two levels: metabolite concentrations and network structure. Alterations in the reaction content of a metabolic network may alter its capacity to produce metabolites, i.e., the maximum theoretical yield of metabolite biosynthesis. Therefore, we used metabolite production capacities, derived from computational models of genome-scale metabolic networks, as measures of how the structure of the entire metabolic network determines its metabolic potentials. Note that metabolite production capacities represent maximum possible biosynthetic yields and therefore can be calculated from the structure and reaction stoichiometries of the metabolic network, without requiring any regulatory or enzyme kinetic information. We leveraged genome-scale metabolic network reconstructions available for 7 yeast species and 54 *S. cerevisiae* strains in our dataset. These networks have been reconstructed using similar methodology based on genomic information and contain on average ~1,100 genes and ~4,000 associated reactions per strain (8, 34).

**Fig. 2.**
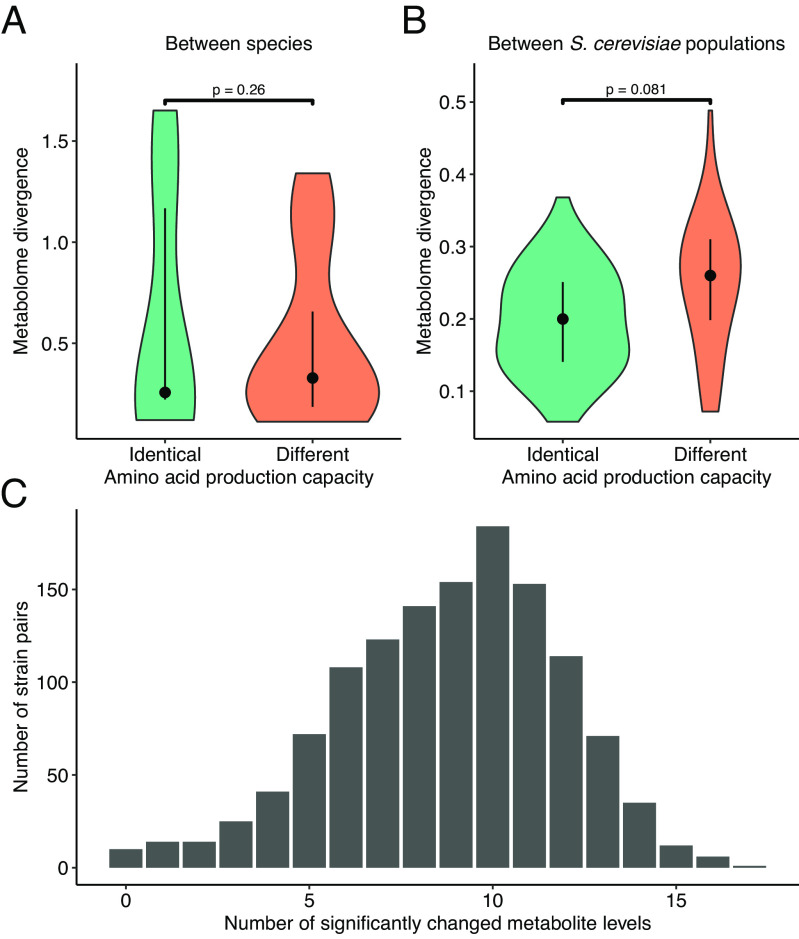
Evolutionary divergence in metabolome profile versus metabolite production capacity profile. (*A*) Amino acid metabolome distance of pairs of yeast species. Species pairs are divided into two groups based on their similarity in amino acid production capacity. Pairs with identical (N = 9) or different (N = 12) production capacities are shown in blue and orange, respectively. The *P*-value was determined using a permutation test (*Methods*). (*B*) Amino acid metabolome distance of *S. cerevisiae* population pairs. Population pairs are divided into two groups based on their similarity in amino acid production capacity. Pairs with identical (N = 91) or different (N = 45) production capacities are shown in blue or orange, respectively. The *P*-value was determined using a permutation test. (*C*) Extent of metabolite variations between *S. cerevisiae* strain pairs with identical amino acid yields. Distribution of the number of significantly changed amino acid levels are shown for those *S. cerevisiae* strain pairs which have no difference in their amino acid yields.

To test whether evolutionary divergence in the overall amino acid synthesis capacity correlates with divergence in the amino acid metabolome, we calculated the profile similarity of computed maximum yields across the 19 amino acids for each pair of yeast species and each pair of *S. cerevisiae* populations (*Methods*). We found that the yield profile similarity follows a binomial distribution both within and between species, with several species pairs and the vast majority of population pairs exhibiting identical amino acid production capacities (*SI Appendix*, Table S2 and Dataset S8). Remarkably, we found no significant difference in the overall divergence of amino acid levels when comparing species pairs with identical versus dissimilar production capacities (Fig. 2*A*, *P* = 0.26, permutation test). In a similar vein, *S. cerevisiae* populations that differ in their amino acid production capacities are not more diverged in their amino acid levels than those with identical production capacities (Fig. 2*B*, *P* = 0.081, permutation test).

In part, the lack of association between divergence in synthesis capacity and metabolite levels might be due to the faster evolution of metabolite levels than network structure. Indeed, the large majority of *S. cerevisiae* strain pairs show identical amino acid production capacities (1,278 out of 1,431 pairs among 54 strains). Remarkably, virtually all (98.6%) such strain pairs show a significant change in at least one amino acid, with a median of 9 significantly altered amino acid levels (Fig. 2*C* and Dataset S9). Further support was provided by strain pairs of *S. cerevisiae* with identical reaction repertoires and, consequently, identical metabolic network structures. Again, we found that such strain pairs have a median difference of 8 amino acid levels (*SI Appendix*, Fig. S2). Thus, even strains with identical metabolic network structures or metabolite production capacities differ substantially in their metabolite levels. Overall, these findings indicate that the evolution of metabolite levels is decoupled from the metabolic network’s structure and occurs at substantially faster rates. This is broadly consistent with the notion that network structure is highly constrained by natural selection, whereas metabolite levels are subject to less stringent selection and accumulate a considerable amount of neutral changes. If so, variation in metabolite levels should largely reflect phylogenetic history. Alternatively, divergence in metabolite levels might represent adaptive differences in metabolic regulation that are not caused by rewired network structures. In the next section, we examine the influence of phylogenetic history and ecological origins on metabolome variation.

### Metabolome Variation Is Driven by Both Population History and Domestication in *S. cerevisiae*.

We next interrogated the temporal dynamics of metabolome divergence by focusing on *S. cerevisiae*, which is represented by 17 genetically differentiated populations in our dataset. Plotting the overall divergence of 19 amino acid levels as a function of phylogenetic distance between pairs of populations revealed no significant correlation between the two (*SI Appendix*, Fig. S3, r = 0.33, *P* = 0.33, phylogenetic Mantel test). This pattern may reflect population-specific changes in metabolism induced by major lifestyle differences among distinct *S. cerevisiae* populations.

**Fig. 3.**
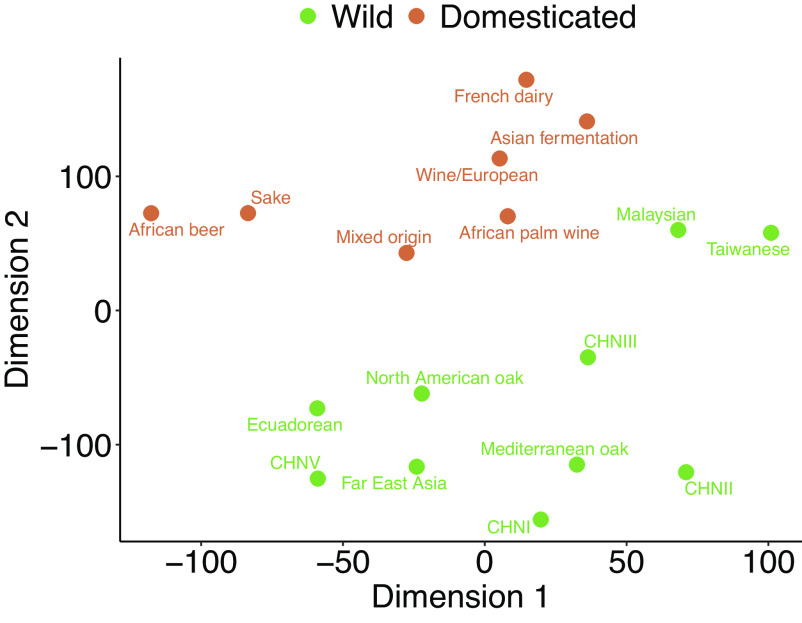
Separation between wild and domesticated *S. cerevisiae* populations in the amino acid metabolome. t-distributed stochastic neighbor embedding (t-SNE) algorithm was applied on the data. Green and orange dots indicate wild and domesticated populations, respectively.

**Fig. 4.**
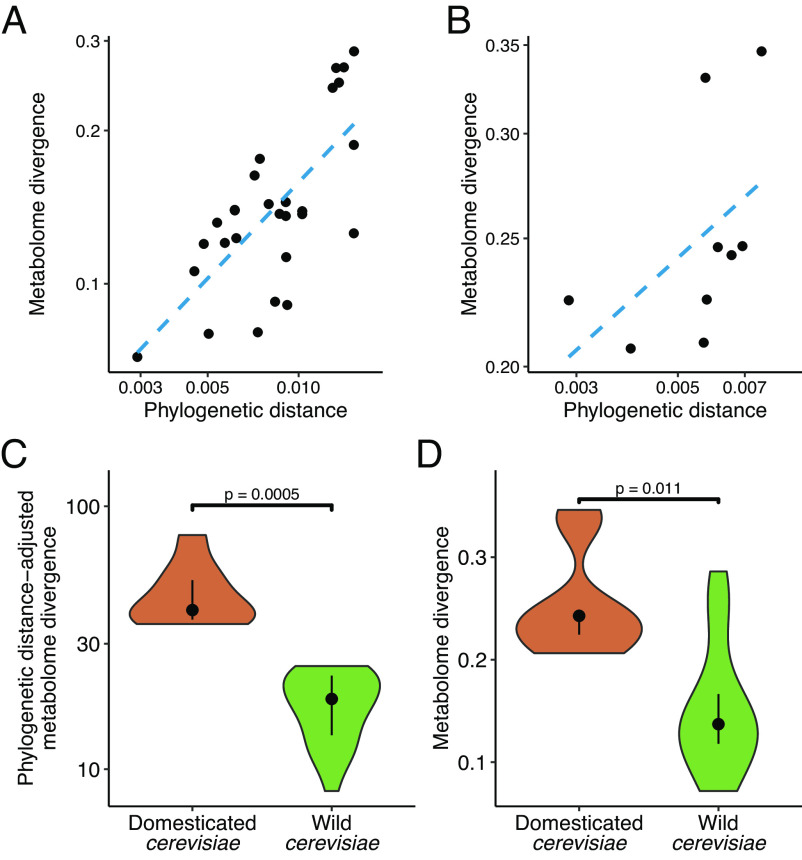
Metabolome divergence as a function of phylogenetic distance among *S. cerevisiae* population pairs based on amino acid measurements. (*A*) Pairs of wild *S. cerevisiae* populations (N = 28). North American oak and Mediterranean oak populations are excluded, as their closest relatives are domesticated populations. The dashed line (blue) indicates the linear regression line. (*B*) Pairs of domesticated populations that come from the same domesticated clades (N = 9). The dashed line (blue) indicates the linear regression line. (*C*) Comparison of phylogenetic distance-adjusted metabolome divergence among pairs of domesticated (N = 9) and wild (N = 28) *S. cerevisiae* populations. The *P*-value was determined using a permutation test (*Methods*). (*D*) Comparison of metabolome divergence among pairs of domesticated (N = 9) and wild (N = 28) *S. cerevisiae* populations. The *P*-value was determined using a permutation test.

Domestication has been identified as the most dramatic life history-changing event during the intra-species evolution of *S. cerevisiae* that profoundly influenced the biology of the domesticated populations (35, 36). We thus hypothesized that domestication also had a major impact on metabolome evolution, overriding the signatures of phylogenetic relatedness among populations. Two lines of evidence support this scenario. First, we found that the overall metabolome profile differs substantially between wild and domesticated populations (Fig. 3 and *SI Appendix*, Fig. S4). Such a difference may indicate adaptation to human-made niches and is analyzed in more detail below. Second, we found that the overall metabolome divergence correlates with phylogenetic distance when wild and domesticated clades are analyzed separately. Specifically, consistent with earlier studies (23, 24), reconstructing the phylogenetic relationship between *S. cerevisiae* populations revealed two widely separated clades containing predominantly domesticated populations, suggesting at least two independent domestication events (*SI Appendix*, Fig. S5). Removing these two domesticated clades revealed a strong correlation between metabolome divergence and phylogenetic distance among wild populations (r = 0.74, *P* = 0.013, phylogenetic Mantel test, Fig. 4*A*). Similarly, metabolome divergence correlates well with phylogenetic distance among pairs of domesticated populations belonging to the same domesticated clades (r = 0.96, *P* = 0.006, phylogenetic Mantel test, Fig. 4*B*). Similar results are obtained when defining overall metabolome divergence based on the global metabolome profile excluding amino acids (r = 0.75, *P* = 0.03 for wild and r = 0.98, *P* = 8.5e-4 for domesticated population pairs, phylogenetic Mantel test, *SI Appendix*, Fig. S6). Thus, metabolome variation largely follows the genetic history of populations in *S. cerevisiae*, with the exception of major metabolome rewiring associated with domestication events.

We next asked whether domesticated and wild populations differ in their tempo of metabolome diversification. To estimate the rates of metabolome evolution, we calculated phylogenetic distance-adjusted metabolome divergences between pairs of wild populations as well as between pairs of domestic populations that come from the same domesticated clades. Remarkably, the phylogenetic distance-adjusted metabolome divergence is ~2.6-fold and 5.7-fold higher among domesticated populations than among wild populations for amino acids and non-amino acid metabolites, respectively, indicating a faster overall tempo of metabolome evolution in domesticated yeasts (Fig. 4*C*; for non-amino acid metabolites see and *SI Appendix*, Fig. S7). In general, evolutionary diversification is slower when measured over longer evolutionary time intervals (4, 37) and our dataset includes some highly diverged wild populations, potentially biasing the above comparison. However, the inferred faster tempo of metabolome evolution in domesticated yeasts is not an artifact of comparing evolutionary rates over very different time scales. First, even without adjusting for phylogenetic distance, domesticated populations show larger metabolome differences than wild populations despite being genetically less diverged from each other (Fig. 4*D*). Second, after excluding wild population pairs that are phylogenetically more distant than any of the domesticated population pairs, we still observe a 2.2-fold higher phylogenetic distance-adjusted metabolome divergence among domesticated populations than among wild populations (*SI Appendix*, Fig. S8). The rapid metabolic diversification of domesticated yeasts is well illustrated by several differences in specific metabolite levels between populations coming from the same domesticated clades (Dataset S10). For example, on average, isolates from the African beer population display higher alanine and proline levels than isolates from the Wine population (*SI Appendix*, Fig. S9).

Finally, while analyzing the dynamics of metabolome evolution across species would require a larger species set, we note that metabolome differences appear to accumulate gradually with phylogenetic distance among species (*SI Appendix*, Fig. S10, r = 0.71 and *P* = 0.011 for amino acids and r = 0.91 and *P* = 4.6e-4 for non-amino acid metabolites, phylogenetic Mantel tests). As this pattern resembles the intra-species variation patterns without domestication, we conclude that phylogenetic history may generally play an important role in metabolome divergence in budding yeasts.

### The Metabolic Domestication Syndrome of *S. cerevisiae*.

The substantial metabolome differences observed between wild and domesticated *S. cerevisiae* populations are consistent with at least two scenarios. First, evolution in human-made niches might result in a set of universal metabolomic changes, i.e., a metabolomic domestication syndrome, regardless of the genetic makeup of the ancestor and the specific details of the domestication niches. Alternatively, populations from the two major and widely separated domesticated clades might differ substantially both from wild yeasts and from each other in their metabolomes due to differences in the genetic makeup of their ancestors and/or selective forces. To distinguish between these scenarios, we systematically compared the levels of individual metabolites across domesticated and wild populations. Specifically, we performed a series of phylogenetic ANOVAs to test i) whether domesticated populations show recurrent metabolite changes compared to wild populations that cannot be explained by shared ancestry alone, and ii) whether populations from the two distinct domesticated clades differ from each other.

**Fig. 5.**
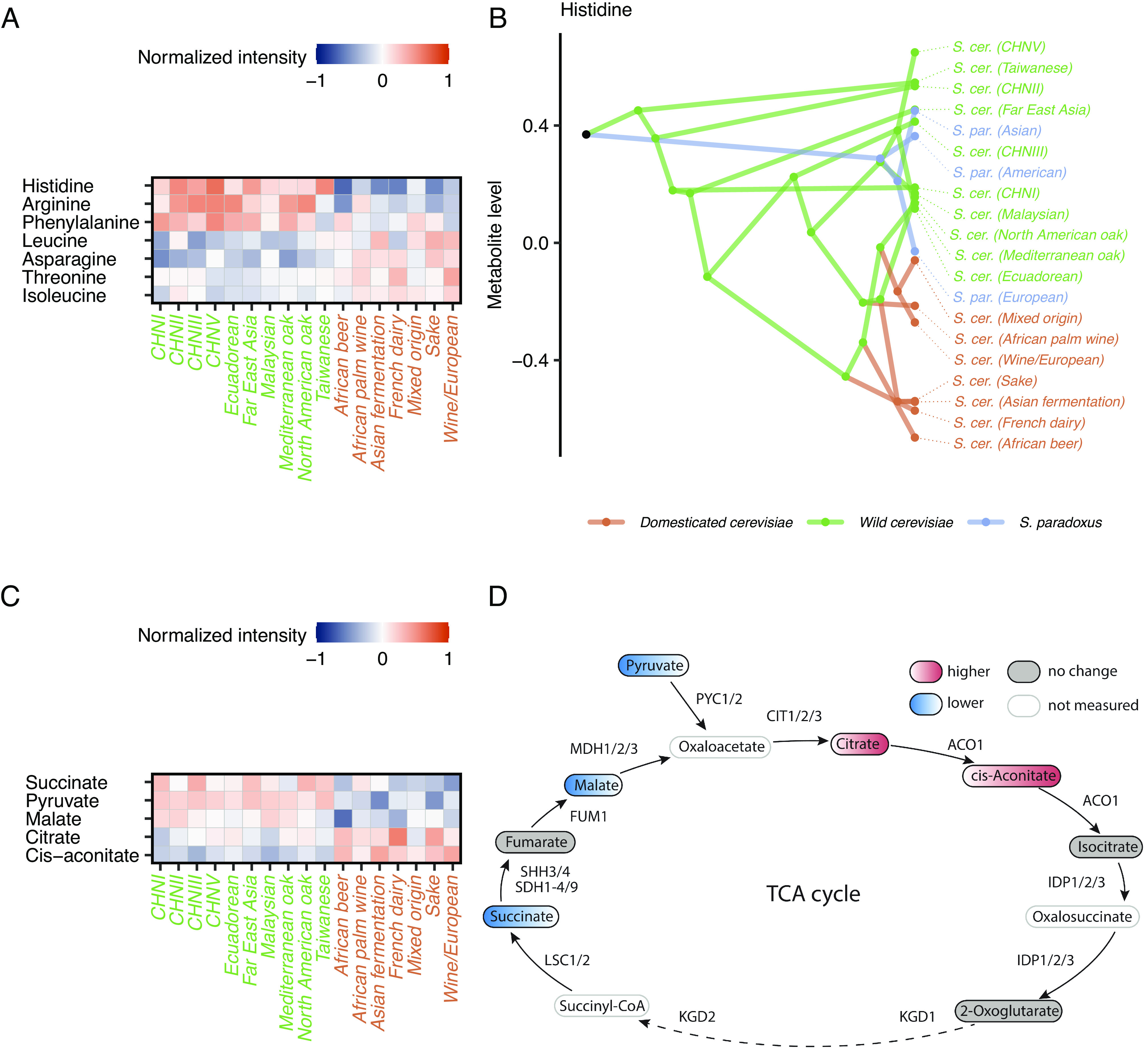
The metabolomic domestication syndrome in *S. cerevisiae* populations. (*A*) Average levels of amino acids showing domestication signature in across *S. cerevisiae* populations. Wild and domesticated populations are marked as green and orange, respectively. Metabolite levels are studentized for each metabolite for visualization purposes. (*B*) Traitgram of histidine level changes on the *S. cerevisiae* phylogenetic tree. Note that branch lengths do not scale with phylogenetic distance for visual clarity. Domesticated, wild *S. cerevisiae*, and *S. paradoxus* populations are colored as orange, green, and blue, respectively. (*C*) Average levels of TCA cycle metabolites showing domestication signature across *S. cerevisiae* populations. Wild and domesticated populations are marked as green and orange, respectively. Metabolite levels are studentized for each metabolite for visualization purposes. (*D*) Schematic of the TCA cycle (based on ref. 38). Metabolites with increased or decreased levels in domesticated populations are marked as red or blue, respectively. Metabolites showing no domestication signature and unmeasured metabolites are denoted with filled gray and white labels, respectively.

Our analysis revealed pervasive signatures of parallel evolution in the metabolomes of domesticated yeast (i.e., domestication signature). Specifically, 7 out of 19 amino acids and 27 out of 78 non-amino acid metabolites display a significantly increased or decreased level in independently domesticated populations compared to wild populations (Fig. 5*A*, *SI Appendix*, Fig. S11, and Dataset S11). For example, histidine displays a particularly strong signature: domesticated populations have, on average, twofold lower histidine levels than wild populations. As *S. paradoxus* populations exhibit similar histidine levels to wild *S. cerevisiae* populations, the low histidine levels of domesticated populations are likely to be derived states and have evolved convergently (Fig. 5*B*).

Several specific amino acids have been implicated in the formation of various aroma compounds (39) and therefore their metabolism might have been shaped by domestication. Consistent with this scenario, four of the seven amino acids (leucine, isoleucine, phenylalanine, and threonine) with a domestication signature also impact aroma production according to a systematic study (39), which is a statistically significant overlap (*P* = 0.038, Fisher’s exact test, Dataset S11).

The set of metabolites displaying parallel changes in domesticated populations covers ~35% of the compounds measured via non-targeted metabolomics and span several metabolic subsystems, indicating a widespread metabolome signature associated with domestication (*SI Appendix*, Fig. S11). Notably, this signature also includes several metabolites of the TCA cycle. To confirm the domestication signature of the TCA cycle, we quantified the levels of TCA intermediates using a well-established targeted metabolomics method (40) (Dataset S3 and *Methods*). Reassuringly, 5 out of the 8 measured TCA intermediates display a domestication signature, with metabolites in the early steps of the TCA cycle having increased, and succinate and malate decreased levels (Fig. 5 *C* and *D*). This pattern indicates that domestication rewired the TCA cycle, possibly as a consequence of adaptation to better utilize carbon sources that support fermentative growth (36).

In sharp contrast to the widespread metabolome rewiring between wild and domesticated yeasts, we found no significant metabolome differences among populations belonging to the two major phylogenetically separated domesticated clades (Dataset S12). Furthermore, despite a marked difference between the current environments of the French dairy strains and the Wine and Sake populations (i.e., Camembert cheese or raw milk versus alcoholic fermentation) the domestication signature is also present in the French dairy population (Fig. 5 *A*–*C*). We note that the domestication signature is also evident when the Asian fermentation population is removed from the analysis (Dataset S11), which is a clade with a less clearly determined domestication status than the other populations (36). In addition, further statistical analysis confirmed that the domestication signature is not an artifact of potential growth state differences between strains at the time of sampling (Dataset S11). Overall, these results suggest that independent domestication events converged on a recurrent metabolome signature, supporting the existence of a universal metabolic domestication syndrome in *S. cerevisiae*.

Last, we asked whether other ecological factors beyond domestication have shaped the yeast metabolome. The *S. cerevisiae* populations studied here originate from a range of geographical and climatic regions. However, we found no evidence that yeast strains isolated from the same climate zone or broad geographic area show increased metabolome similarities (*SI Appendix*, Tables S3 and S4).

## Discussion

In this paper, we combined metabolomic measurements with phylogenetic comparative analyses to study the evolution of metabolic states in the unicellular model eukaryote budding yeast. By studying variation of central metabolites across a panel of natural isolates representing the major clades of *S. cerevisiae* and several related species, we reached several general conclusions.

We found that although metabolite levels diverge mostly gradually with evolutionary time, domestication has impacted them profoundly. Domestication had major effects on the genome, life history traits, and nutrient utilization capacity of *S. cerevisiae* (35, 36). These traits are shared between phylogenetically separated and independently domesticated lineages, indicating convergent evolution of domestication-associated traits and hence the existence of a yeast domestication syndrome. Our results demonstrate that a substantial fraction of metabolite levels also evolved convergently in independently domesticated clades, revealing a recurrent metabolic domestication syndrome in yeast. The set of affected metabolites covers various central pathways from amino acid metabolism to pyruvate metabolism and the TCA cycle. For instance, several metabolites of the TCA cycle have altered levels in domesticated populations, possibly as a consequence of adaptation to fermentative growth (36). We also found evidence for the convergent evolution of several amino acid levels across distinct domesticated clades. Since the biosynthesis of several amino acids originates from the TCA cycle, one might expect an overlap between these amino acids and those with domestication signatures. However, visual inspection revealed no clear link between the two sets (*SI Appendix*, Fig. S13). Consequently, parallel changes in amino acid levels and TCA cycle intermediates cannot simply be explained by their topological proximity in the network. A previous study reported that two of the domestication-associated amino acids (threonine and isoleucine) are better utilized as nitrogen sources by domesticated than wild yeasts (36). This indicates that domestication changed both the intracellular levels and consumption of these amino acids. Most notably, four out of seven domestication-associated amino acids (leucine, isoleucine, phenylalanine, and threonine) impact aroma production (39), suggesting that their evolution may have been driven by human selection on aroma profiles. Indeed, a previous study reported convergent evolution of a specific aroma compound across multiple domesticated lineages (19). Overall, our study expands such case studies and indicates pervasive rewiring of the metabolome upon adaptation to human-made niches. Thus, domestication reprogrammed not only the life cycle of yeast (36) but also its central metabolic pathway activities. This finding has far-reaching implications for our understanding of domestication in this prime model organism.

An important open question is whether analogous signatures of domestication are also apparent at the gene expression level and whether the metabolomic signature is primarily driven by gene regulatory changes. Previous studies of transcriptome diversity across *S. cerevisiae* isolates have not explicitly sought to identify a recurrent signature of domestication, so there is room for alternative interpretations. On the one hand, it appears that most transcriptomic variation follows the phylogenetic history (41) and is dominated by population-specific transcriptional signatures, including those that are unique to each domestication (42). However, these studies do not rule out the possibility of a common transcriptional signature of domestication that involves specific gene sets. Indeed, an earlier study reported recurrent gene expression changes in the TCA cycle and fermentation pathways between isolates from the Wine and Sake populations (25). More in-depth analyses are required to unravel the possible gene regulatory changes linked to the metabolic domestication syndrome.

Our findings have implications for the neutral theory of molecular evolution. According to this theory, most variations in nucleotide and protein sequences within and between species are selectively neutral (43). Although the theory was specifically proposed to explain sequence evolution, in principle, it could be also applied to molecular traits that are not perfectly correlated with organismal fitness (44). Indeed, gene expression levels appear to evolve largely neutrally in both yeasts and animals (41, 45, 46). However, it is largely unclear whether molecular traits that lie closer to fitness, such as metabolite levels, are mainly governed by adaptive or neutral evolution. We found that phylogenetic relatedness explains well the overall divergence of metabolite levels among *S. cerevisiae* populations after accounting for domestication (Fig. 4). This pattern is broadly consistent with a neutral scenario, where metabolome variations arise largely due to the steady accumulation of neutral or nearly neutral mutations. Notably, similar results have been reported for variation in gene expression and several phenotypic traits in *S. cerevisiae* (41, 47). The neutral scenario is further supported by our observation that, beyond domestication, *S. cerevisiae* isolates originating from similar environments do not show increased similarity in their metabolomes. In addition, our finding that different amino acids diverge at different rates also fits into the neutral theory. Specifically, we found that metabolites whose levels vary more among species also tend to show larger variations across *S. cerevisiae* populations (Fig. 1 *B* and *C*). This pattern is consistent with the notion that some metabolites are subject to less stringent selective constraints and therefore can accumulate more neutral changes across multiple evolutionary timescales. Recent research into the evolution of metabolite levels in mammals found similar results. It has been shown that evolutionary conservation of metabolite levels varies greatly among metabolites and can be explained by a neutral model (22).

Despite such evidence supporting neutral evolution, a substantial part of the metabolome (>30% of measured metabolites) shows signatures of convergent evolution across independently domesticated lineages, indicating that adaptive evolution has also substantially shaped the metabolome diversity of *S. cerevisiae*. Adaptive evolution has also been shown to be responsible for metabolite changes associated with wheat domestication (15). Clearly, the relative importance of neutral versus adaptive evolution of metabolite levels in yeasts remains to be formally quantified, for example, through quantitative genetic methods (15). In addition, further study is needed to test whether the faster rate of metabolome divergence among domesticated populations is driven by niche adaptations or enhanced drift owing to population bottlenecks.

We found that metabolite levels evolve faster than the metabolic network’s capacity to produce metabolites (i.e., yield). Notably, closely related yeast isolates often show identical or highly similar enzyme reaction repertoires, yet display substantial metabolome differences. More broadly, the evolution of metabolic states appears to be largely uncoupled from divergence in the structure of the network. Evolution at these two levels might differ for several reasons. First, the underlying mutational target sizes differ greatly. While the rewiring of metabolic pathway structure depends on highly specific gene gain and loss events, metabolite levels can be impacted by a broad range of mutations. For example, individual amino acid levels are influenced by the activity of up to ~400 genes in yeast (13). Thus, mutations in many proteins that do not directly interact with a metabolite can also affect its level. Second, metabolic reaction repertoires are likely under strong purifying selection with limited room for selectively neutral changes. Indeed, the vast majority of genes, including enzyme-encoding ones, have a measurable fitness contribution under some environmental conditions (48, 49). Thus, phylogenetic variation in enzyme repertoire likely reflects environmental adaptation. In contrast, metabolite levels may readily vary without measurably affecting fitness (20, 21) and may therefore evolve more rapidly and independently of adaptive changes in the network’s structure. Based on these considerations, we propose that studying metabolic evolution through metabolomics provides largely complementary information that cannot be obtained by studying enzyme content alone. This view extends recent findings that patterns of natural selection on metabolite levels are not easily predictable from selection acting on gene expression (50).

Our study has several limitations. First, the examined strain panel captures divergence between clean (i.e., non-mosaic) *S. cerevisiae* populations and hence is well suited for phylogenetic comparative analyses. However, our study was not designed to investigate within-population diversity or evolutionary changes in mosaic strains. Second, the metabolome measurements were performed in a single environment that is ideally suited for assessing variation in biosynthetic metabolism (13) and in which all strains exhibit stable growth. This corresponds to the common garden design in comparative metabolomics studies (51). However, in the real world, yeasts experience a plethora of environmental conditions and therefore our study likely underestimates the variety of metabolomic states displayed by different lineages. Importantly, the metabolomic signature of domestication is evident in all human-associated populations regardless of ecological differences. It is therefore unlikely that the domestication signature is an artifact of measuring metabolomes in a single environment to which some domesticated clades are better adapted than others. Nevertheless, we note that measuring phylogenetically diverged species in the same environment can be especially challenging as they might display very different physiology. Indeed, *S. castelli* displays the lowest growth rate among the examined species (Dataset S1), which may confound its perceived metabolome divergence. Finally, our study only included single strains of several species, which may limit the accurate estimation of metabolome differences between species. In light of the substantial metabolic diversity observed even within wild *S. cerevisiae*, studying metabolome variation in other yeast species is an important open question.

In sum, our work uncovers substantial variations in central metabolite levels across budding yeasts and establishes a recurrent metabolomic signature of domestication.

## Methods

### Strains.

*S. cerevisiae* isolates included in this project were sequenced and analyzed previously (52) and provided by Gianni Liti. Strains were carefully selected to be representative of non-admixed wild and domesticated *S. cerevisiae* populations with at least three strains when possible. *S. paradoxus*, *Saccharomyces mikatae*, and *Saccharomyces kudriavzevii* isolates were selected from the *Saccharomyces* Genome Resequencing Project (SGRP) collection and sequenced and analyzed previously (52). *Saccharomyces arboricola, Saccharomyces eubayanus, S. castelli*, and *Kazachstania saulgeensis* strains were purchased from CBS (https://wi.knaw.nl/Collection). The *Saccharomyces jurei* isolate was provided by Daniela Delneri.

### Growth Rate Measurements.

We performed growth rate measurements at both 25 °C and 30 °C to select a temperature that minimizes growth rate differences across the diverse yeast isolates studied here. Yeast cells were inoculated from frozen (−80 °C) samples on 2% agar solidified synthetic dextrose (SD) medium (0.145%yeast nitrogen base minus amino acids/0.5% ammonium sulfate, and 2% glucose + 2% agar). Cells were cultivated for 48 h at 25 °C and 30 °C. Pre-cultures were inoculated from colonies in 96-well format using Hamilton Microlab star liquid handling robot equipped with core grippers and V&P scientific pin-tool with 1.58-mm floating pins (53). Pre-cultures were cultivated in 96-well plates in SD media for 16 h using a Heidolph Titramax 1000 (900 rpm) vibrating plate shaker incubator at both temperatures.

We used a protocol designed to measure growth rate in minimal media (53). Growth was assayed by monitoring the OD (OD_600_) of liquid cultures of each strain using 384 well microtiter plates (Corning). The 384-well plates filled with 60 µL medium per well were inoculated for growth curve recording with 1% starter culture. The 384-well plates were incubated at 25 °C with medium linear shaking in Powerwave HT plate readers (BioTek Instruments Inc). Cell growth was followed by recording the OD at 600 nm every 5 min. Four technical replicate measurements were executed on all strains. Growth rate was calculated from time series OD_600_ data as described elsewhere (47).

### Targeted Quantification of Amino Acids.

For metabolomics experiments, pre-cultures were cultivated as described for growth rate measurements. We inoculated 4% pre-cultures into 1,200 µL/wells of SD media in a 2 mL/well deep well plate and cultivated for 10 h at 25 °C (until the mid-exponential phase) in a Heidolph Titramax 1000 vibrating plate shaker incubator at 900 rpm. Stirring was enhanced by using a 2-mm size borosilicate glass/well. To obtain OD values at the time of inoculation, sampling, and stationary phase, we cultivated a separate plate in parallel and took a sample volume of 100 µL for OD measurement at the time of sample harvest for metabolome measurement. Growth state was calculated by subtracting OD values at inoculation from OD values at sampling and dividing this result by the OD values at stationary phase (Dataset S1). ODs were measured using a Powerwave HT plate reader. The full sample was harvested and extracted as described in ref. 13 with minor modifications. Briefly, cell pellets were extracted with 200 µL 80 °C hot ethanol containing isotope labeled algal extract 5 mg/mL extraction solution. We obtained Algal Lyophilized Cells (U-13C, 98%+) from Cambridge Isotope Laboratories CLM-2065-PK. After 1 min of vigorous vortexing, we let the mixture incubate without shaking for 5 min and centrifuged for 5 min at maximum rpm. The aqueous phase was transferred to the prepared extraction solution. LC–MS-based quantification was also carried out in the same way as described in ref. 13, but free amino acids of ~0.5 mg 13C-labeled algae/sample extracts were used as internal standard for quantification instead of algal hydrolysate amino acid mixture. Metabolite identification was supported with standard injection and by monitoring 1 quantifier + 1 qualifier ion/metabolite. Metabolic concentration data were normalized using i) probabilistic quotient normalization (PQN) to control for biomass amount and extraction efficiency differences between replicates and ii) linear regression between OD at the time of sampling and metabolite intensities to control for potential intensity differences stemming from growth differences (32).

### Preparation of Internal Standards for Amino Acid Quantification.

13C-labeled algae were extracted using a modified Bligh-and-Dyer extraction method (54). First, 700 µL of 4:10 water/MetOH was added to the 250 mg of lyophilized algal cells and vortexed vigorously. It was followed by the addition of 250 µL chloroform and vortexed vigorously for 1 min. After this, 250 µL water was added and vortexed vigorously for 1 min, which was followed by the addition of 250 µL chloroform again.

### Non-Targeted Metabolomics.

For non-targeted metabolomics, 1,200 µL culture/sample was cultivated at 25 °C in the same way as for amino acid quantification. For metabolite extraction, 400 µL of cells/sample were centrifuged at 4 °C for 3 min (13). Cell pellets were extracted using 200 µL of 40:40:20 (v/v/v) methanol/acetonitrile/water (55, 56) with 900 rpm shaking for 10 min at 4 °C. After pelleting the cells with 4,500 rpm −20 °C centrifugation for 5 min, the cells were re-extracted with the same method. Combined extracts from both extractions were centrifuged to remove debris. A pooled extract (QC, quality control) was prepared by joining the extracts from each experimental batch separately. Extracts were stored at −80 °C for high resolution mass spectrometry (HR-MS) measurements using polypropylene plates with a V-bottom. Before analysis, extracts were thawed and shaken for 10 min at room temperature at 900 rpm with Titramax 101. Metabolite extracts (2 µL) were injected directly into a Thermo Q-exactive Focus mass spectrometer (Thermo Fisher Corporation) operated in negative mode only, at 70,000 resolution, 2 micro-scans, and 3 × 10^6 AGC, using Dionex 3000 HPLC eluent flow. Flow rate was 150 mL/min, and the mobile phase was acetonitrile:water (70:30, v/v). To ensure measurement stability during analysis, a pooled extract sample (QC) was assessed every 20 samples (57). We used ProFIA, a data preprocessing workflow specifically designed to process high-resolution, high-throughput metabolomics data for flow injection analysis (58). Signal to noise threshold was set to 10 and background intensities were measured at the last 30 s of the 3-min runtime. In one experimental batch, 4 replicates were cultivated and measured.

We assigned putative metabolite to the measured ions using a high-quality genome-scale reconstruction of yeast metabolism (34, 59) based on their exact masses and only considering deprotonation. Putative metabolites were kept only if the i) corresponding ion was detected in at least 87.5% of the pooled extract (QC) samples, which were injected 8 times at the beginning of the measurement sequence, ii) showed a CV (coefficient of variation) of peak area lower than 25% and iii) was detected in at least 90% of the biological samples. Missing values were imputed using the k-nearest neighbors method (60, 61). Intensities were normalized as in amino acid quantification, i.e., using PQN normalization and linear regression between OD at the time of sampling and metabolite intensities (32).

### Targeted Quantification of TCA Cycle Intermediates and Pyruvic Acid.

We followed an established method (40) with small modifications for the quantification of TCA cycle intermediates and pyruvate. We determined TCA cycle intermediates and pyruvic acid from 1,000 µL culture/sample in deep well plates. Pellets were extracted in 40:40:20 (v/v/v) methanol/acetonitrile/water and dried. Dried extracts were dissolved in 150 µL LC–MS grade water and cleared. Compounds were separated using a Waters HSS T3 column (1.7 μm, 2.1 mm × 100 mm) on a liquid chromatography (Waters ACQUITY Premier) and tandem mass spectrometry (Waters TQS-Micro) system. For a more detailed description, see *SI Appendix*.

### Phylogenetic Analyses.

We constructed a phylogenetic tree of the budding yeasts studied here using a phylogenomic approach following (62). We used the genomes of the 71 yeast isolates and two outgroups, *Torulaspora delbrueckii CBS1146,* and *Tetrapisispora phaffii CBS4417*. Genome sequences were acquired from different sources, see Dataset S6, column “Sequence source” (23, 28, 35, 52, 63–67). To obtain consistent genome annotation across the studied yeast isolates, we annotated all budding yeast genomes, except the outgroups, using the MAKER genome annotation pipeline v2.31.10 (68). For a detailed description, see *SI Appendix, Supporting text, Genome Annotation* section. Note that for the outgroups, we used the published genome annotations.

To construct the phylogenetic tree, we first translated all nucleotide sequences to protein sequences with the transeq tool from the EMBOSS package (version 6.6.0) (69). Next, we used OrthoFinder (version 2.4.0) (70) to cluster homologous genes across all genomes. The clustering resulted in 1,1280 orthogroups in total. Of note, 1,218 orthogroups contained a single gene copy from each genome and were further considered for concatenation-based phylogenetic tree inference. We performed multiple sequence alignment on each of the 1,218 orthogroups using MAFFT (version 7.471) (71). TrimAl (version 1.4.1) (72) was applied with the “-gappyout” option to remove poorly aligned regions. Trimmed sequences shorter than 167 amino acids or shorter than 50% of the length of the total trimmed alignment were removed. We also removed sequences with missing amino acids. Orthogroups containing removed sequences were excluded from further analysis. After filtering, we concatenated the remaining 983 orthogroups to generate a single concatenated sequence for each genome. We built a concatenated maximum likelihood tree with RAxML (AVX version, 8.2.12) (73) using the LG amino acid substitution matrix with the GAMMA model and using one partition for each orthogroup. Rapid bootstrapping was enabled, and the “autoMRE” option was used to determine the sufficient number of bootstrap replicates. Most internal nodes at or above the population level received bootstrap support values higher than 95% (*SI Appendix*, Fig. S12). The tree with the best likelihood value was collected to represent the phylogenetic relationships between the strains in our dataset (Dataset S4). However, most of our analyses focus on the phylogenetic relationships of yeast populations instead of individual strains. In order to obtain a phylogenetic tree of populations, we selected one representative strain from each population and removed the rest of the strains from the tree (Dataset S5).

### Calculation of Metabolome Divergence.

We calculated metabolome divergence between strain pairs as the average of the squared differences in metabolite levels for each metabolite (or putative metabolite in the case of the non-targeted dataset) using the following equation:

$$\frac{\sum_{i=1}^{N}{\left(m_{k,i}-m_{l,i}\right)}^{2}}{N},$$

where *N* is the number of metabolites in the dataset, *k* and *l* represent two separate strains, and *m_k,i_* and *m_l,i_* represent the log-scaled and OD normalized level of the *i*th metabolite in strain *k* and *l*, respectively. Metabolome divergence between a pair of population was calculated as the average metabolome divergence of strain pairs where each strain belongs to one of the two populations. Similarly, the metabolome divergence between two species is the average metabolome divergence between strains of the two species.

To calculate phylogenetic distance-adjusted metabolome divergence, we divided the metabolome divergence of each population pair with the phylogenetic distance between the two populations. The phylogenetic distance between two populations is the sum of the lengths of the branches of the phylogenetic tree that connect the two populations to their common ancestor.

### Metabolic Network Structure and Metabolite Production Capacities.

Metabolic network reconstructions and amino acid production capacity (i.e., maximum theoretical yield of metabolite production) simulations were taken from the literature for seven of the species (*S. castelli, S. arboricola, S. cerevisiae, S. eubayanus, S. kudriavzevii, S. mikatae, and S. paradoxus*) and all of the *S. cerevisiae* strains present in our dataset (Dataset S6). Metabolite yield data were retained for the 19 amino acids present in our dataset. Similarity between yield profiles was calculated in the same way as metabolite divergence; see section “Methods/Calculation of metabolome divergence” for more details. Briefly, for each species/population pair, we calculated the average of the squared differences of metabolite yields for each amino acid. Yield profiles were considered identical when yield distance between species/populations was lower than 1e-3, as small yield differences may occur due to the finite precision arithmetic used in yield calculations. Because several species pairs and the majority of population pairs display identical amino acid yield profiles according to this definition, we compared the metabolome divergence between species and population pairs with identical versus different yield profiles (Fig. 2).

### Statistical Analyses.

#### Phylogenetic mantel test.

We applied phylogenetic Mantel tests to calculate correlations between distance matrices, such as metabolome divergence and phylogenetic distance (74). This method uses phylogeny-based permutations to adjust the *P*-value for the non-independent nature of the values in distance matrices. The strength of the correlation was measured with Pearson’s r. The number of permutations was 1e-5.

#### Permutation test to compare metabolome divergence.

To compare metabolome divergence or phylogenetic distance-adjusted metabolome divergence between different groups of population pairs or species pairs, we employed permutation tests. First, we sorted each population (or species) into groups according to our analysis (e.g., domesticated and wild). Next, we shuffled (resampled without replacement) the group associations of the populations (or species). We calculated the average (phylogenetic distance-adjusted) metabolome divergence of population (or species) pairs for each group and used the ratio of these two numbers as our test statistic. We repeated this randomization process 1e-5 times to acquire a distribution of our randomized test statistic. Finally, we calculated the probability that the original, unshuffled test statistic (ratio of average metabolome divergences between the two groups) comes from our randomized distribution with the following equation:

$$\begin{array}{c} p=\;\frac{(Number\;of\;randomized\;ratios\;higher\;than\;or\;equal\;to\;the\;ratio\;of\;the\;original\;data)\;+1}{(Number\;of\;permutations)\;+\;1}. \end{array}$$

We reported this probability as the p-value of our permutation test. Note that ratios were calculated in a way that the group in the numerator had higher average metabolome divergence in the original, unshuffled data compared to the group in the denominator.

#### Phylogenetic ANOVA.

To determine parallel changes in metabolite levels in domesticated *S. cerevisiae* populations compared to wild populations (or populations with different climates or geographic locations), we performed phylogenetic generalized least squares ANOVA tests (75). First, we calculated the average metabolite levels for each population and marked each population as either wild or domesticated. Then, we used the corBrownian function from the ape package (version 5.7-1) (76) to calculate covariance for the phylogenetic tree of the populations. Next, we used the gls function from the nlme package (version 3.1-162) (77) to fit phylogenetic ANOVA with the average metabolite level and domestication status of each population as variables and the corBrownian object as the correlation structure of the model. We repeated the calculations for each metabolite. The analysis was repeated without the Asian Fermentation population.

In order to control for growth state, we added the average growth state of each population as a covariate to the phylogenetic ANOVA model using the gls function. For each metabolite, the significance level of the independent effect of domestication on the metabolite level was returned.

## Supplementary Material

Appendix 01 (PDF)

Dataset S01 (XLSX)

Dataset S02 (XLSX)

Dataset S03 (XLSX)

Dataset S04 (GZ)

Dataset S05 (GZ)

Dataset S06 (XLSX)

Dataset S07 (XLSX)

Dataset S08 (XLSX)

Dataset S09 (XLSX)

Dataset S10 (XLSX)

Dataset S11 (XLSX)

Dataset S12 (XLSX)

## Data Availability

Raw LC–MS data reported in this article are accessible via Zenodo (https://zenodo.org/records/10680639) under accession number MTBLS9200 (78). Previously published data were used for this work (We used publicly available genome sequence data for yeast isolates from ref. 23).

## References

[1] M. A. Huynen, T. Dandekar, P. Bork, Variation and evolution of the citric-acid cycle: A genomic perspective. Trends Microbiol. **7**, 281–291 (1999). 10390638 10.1016/s0966-842x(99)01539-5

[2] S. Ma , Organization of the mammalian metabolome according to organ function, lineage specialization, and longevity. Cell Metab. **22**, 332–343 (2015). 26244935 10.1016/j.cmet.2015.07.005 PMC4758382

[3] S. Christen, U. Sauer, Intracellular characterization of aerobic glucose metabolism in seven yeast species by 13C flux analysis and metabolomics. FEMS Yeast Res. **11**, 263–272 (2011). 21205161 10.1111/j.1567-1364.2010.00713.x

[4] G. Plata, C. S. Henry, D. Vitkup, Long-term phenotypic evolution of bacteria. Nature **517**, 369–372 (2015). 25363780 10.1038/nature13827

[5] R. Milo, R. L. Last, Achieving diversity in the face of constraints: Lessons from metabolism. Science **336**, 1663–1667 (2012). 22745419 10.1126/science.1217665

[6] T. Yamada, P. Bork, Evolution of biomolecular networks—Lessons from metabolic and protein interactions. Nat. Rev. Mol. Cell Biol. **10**, 791–803 (2009). 19851337 10.1038/nrm2787

[7] B. Papp, R. A. Notebaart, C. Pál, Systems-biology approaches for predicting genomic evolution. Nat. Rev. Genet. **12**, 591–602 (2011). 21808261 10.1038/nrg3033

[8] H. Lu , Yeast metabolic innovations emerged via expanded metabolic network and gene positive selection. Mol. Syst. Biol. **17**, e10427 (2021). 34676984 10.15252/msb.202110427 PMC8532513

[9] T. Y. Pang, M. J. Lercher, Each of 3,323 metabolic innovations in the evolution of E. coli arose through the horizontal transfer of a single DNA segment. Proc. Natl. Acad. Sci. U.S.A. **116**, 187–192 (2019). 30563853 10.1073/pnas.1718997115 PMC6320504

[10] C. D. Herring , Comparative genome sequencing of Escherichia coli allows observation of bacterial evolution on a laboratory timescale. Nat. Genet. **38**, 1406–1412 (2006). 17086184 10.1038/ng1906

[11] S. R. Hackett , Systems-level analysis of mechanisms regulating yeast metabolic flux. Science **354**, aaf2786 (2016). 27789812 10.1126/science.aaf2786 PMC5414049

[12] D. C. Sévin, A. Kuehne, N. Zamboni, U. Sauer, Biological insights through nontargeted metabolomics. Curr. Opin. Biotechnol. **34**, 1–8 (2015). 25461505 10.1016/j.copbio.2014.10.001

[13] M. Mülleder , Functional metabolomics describes the yeast biosynthetic regulome. Cell **167**, 553–565.e12 (2016). 27693354 10.1016/j.cell.2016.09.007 PMC5055083

[14] T. Fuhrer, M. Zampieri, D. C. Sévin, U. Sauer, N. Zamboni, Genomewide landscape of gene–metabolome associations in Escherichia coli. Mol. Syst. Biol. **13**, 907 (2017). 28093455 10.15252/msb.20167150 PMC5293155

[15] R. Beleggia , Evolutionary metabolomics reveals domestication-associated changes in tetraploid wheat kernels. Mol. Biol. Evol. **33**, 1740–1753 (2016). 27189559 10.1093/molbev/msw050 PMC4915355

[16] J. S. Breunig, S. R. Hackett, J. D. Rabinowitz, L. Kruglyak, Genetic basis of metabolome variation in yeast. PLoS Genet. **10**, e1004142 (2014). 24603560 10.1371/journal.pgen.1004142 PMC3945093

[17] G. Zhu , Rewiring of the fruit metabolome in tomato breeding. Cell **172**, 249–261.e12 (2018). 29328914 10.1016/j.cell.2017.12.019

[18] L. Caspeta , Altered sterol composition renders yeast thermotolerant. Science **346**, 75–78 (2014). 25278608 10.1126/science.1258137

[19] B. Gallone , Domestication and divergence of Saccharomyces cerevisiae beer yeasts. Cell **166**, 1397–1410.e16 (2016). 27610566 10.1016/j.cell.2016.08.020 PMC5018251

[20] J. C. Ewald, T. Matt, N. Zamboni, The integrated response of primary metabolites to gene deletions and the environment. Mol. BioSyst. **9**, 440–446 (2013). 23340584 10.1039/c2mb25423a

[21] L. M. Raamsdonk , A functional genomics strategy that uses metabolome data to reveal the phenotype of silent mutations. Nat. Biotechnol. **19**, 45–50 (2001). 11135551 10.1038/83496

[22] O. Liska , Principles of metabolome conservation in animals. Proc. Natl. Acad. Sci. U.S.A. **120**, e2302147120 (2022). 10.1073/pnas.2302147120 PMC10468614 37603743

[23] J. Peter , Genome evolution across 1,011 Saccharomyces cerevisiae isolates. Nature **556**, 339–344 (2018). 29643504 10.1038/s41586-018-0030-5 PMC6784862

[24] J. C. Fay, J. A. Benavides, Evidence for domesticated and wild populations of Saccharomyces cerevisiae. PLoS Genet. **1**, e5 (2005). 16103919 10.1371/journal.pgen.0010005 PMC1183524

[25] D. A. Skelly , Integrative phenomics reveals insight into the structure of phenotypic diversity in budding yeast. Genome Res. **23**, 1496–1504 (2013). 23720455 10.1101/gr.155762.113 PMC3759725

[26] J.-L. Legras , Adaptation of S. cerevisiae to fermented food environments reveals remarkable genome plasticity and the footprints of domestication. Mol. Biol. Evol. **35**, 1712–1727 (2018). 29746697 10.1093/molbev/msy066 PMC5995190

[27] X.-X. Shen , Reconstructing the backbone of the Saccharomycotina yeast phylogeny using genome-scale data. G3 (Bethesda) **6**, 3927–3939 (2016). 27672114 10.1534/g3.116.034744 PMC5144963

[28] S. Naseeb , Whole genome sequencing, de novo assembly and phenotypic profiling for the new budding yeast species Saccharomyces jurei. G3 (Bethesda) **8**, 2967–2977 (2018). 30097472 10.1534/g3.118.200476 PMC6118302

[29] D. P. Bendixsen, N. Gettle, C. Gilchrist, Z. Zhang, R. Stelkens, Genomic evidence of an ancient East Asian divergence event in wild Saccharomyces cerevisiae. Genome Biol. Evol. **13**, evab001 (2021). 33432360 10.1093/gbe/evab001 PMC7874999

[30] T. J. Lee , Extensive sampling of Saccharomyces cerevisiae in Taiwan reveals ecology and evolution of predomesticated lineages. Genome Res. **32**, 864–877 (2022). 35361625 10.1101/gr.276286.121 PMC9104698

[31] P. de Villemereuil, O. E. Gaggiotti, M. Mouterde, I. Till-Bottraud, Common garden experiments in the genomic era: New perspectives and opportunities. Heredity **116**, 249–254 (2016). 26486610 10.1038/hdy.2015.93 PMC4806574

[32] M. Zampieri , High-throughput metabolomic analysis predicts mode of action of uncharacterized antimicrobial compounds. Sci. Transl. Med. **10**, eaal3973 (2018). 29467300 10.1126/scitranslmed.aal3973 PMC6544516

[33] D. Holbrook-Smith, S. Durot, U. Sauer, High-throughput metabolomics predicts drug–target relationships for eukaryotic proteins. Mol. Syst. Biol. **18**, e10767 (2022). 35194925 10.15252/msb.202110767 PMC8864444

[34] H. Lu , A consensus S. cerevisiae metabolic model Yeast8 and its ecosystem for comprehensively probing cellular metabolism. Nat. Commun. **10**, 1–13 (2019). 31395883 10.1038/s41467-019-11581-3 PMC6687777

[35] J.-X. Yue , Contrasting evolutionary genome dynamics between domesticated and wild yeasts. Nat. Genet. **49**, 913–924 (2017). 28416820 10.1038/ng.3847 PMC5446901

[36] M. De Chiara , Domestication reprogrammed the budding yeast life cycle. Nat. Ecol. Evol. **6**, 448–460 (2022). 35210580 10.1038/s41559-022-01671-9

[37] L. J. Harmon , Causes and consequences of apparent timescaling across all estimated evolutionary rates. Annu. Rev. Ecol. Evol. Syst. **52**, 587–609 (2021).

[38] D. E. Metzler, Biochemistry: The Chemical Reactions of Living Cells (Academic Press, ed. 2, 2003).

[39] S. Fairbairn, A. McKinnon, H. T. Musarurwa, A. C. Ferreira, F. F. Bauer, The impact of single amino acids on growth and volatile aroma production by Saccharomyces cerevisiae strains. Front. Microbiol. **8**, 2554 (2017). 29312237 10.3389/fmicb.2017.02554 PMC5742263

[40] O. Al Kadhi, A. Melchini, R. Mithen, S. Saha, Development of a LC-MS/MS method for the simultaneous detection of tricarboxylic acid cycle intermediates in a range of biological matrices. J. Anal. Methods Chem. **2017**, 5391832 (2017). 29075551 10.1155/2017/5391832 PMC5624170

[41] J.-R. Yang, C. J. Maclean, C. Park, H. Zhao, J. Zhang, Intra and Interspecific Variations of Gene Expression Levels in Yeast Are Largely Neutral: (Nei Lecture, SMBE 2016, Gold Coast). Mol. Biol. Evol. **34**, 2125–2139 (2017). 28575451 10.1093/molbev/msx171 PMC5850415

[42] E. Caudal , Pan-transcriptome reveals a large accessory genome contribution to gene expression variation in yeast. bioxiv [Preprint] (2023). 10.1101/2023.05.17.541122 (Accessed 28 July 2023). PMC11176082 38778243

[43] M. Kimura, The Neutral Theory of Molecular Evolution (Cambridge University Press, 1983), 10.1017/CBO9780511623486.

[44] J. Zhang, Neutral theory and phenotypic evolution. Mol. Biol. Evol. **35**, 1327–1331 (2018). 29659993 10.1093/molbev/msy065 PMC5967557

[45] J. Chen , A quantitative framework for characterizing the evolutionary history of mammalian gene expression. Genome Res. **29**, 53–63 (2019). 30552105 10.1101/gr.237636.118 PMC6314168

[46] P. Khaitovich , A neutral model of transcriptome evolution. PLoS Biol. **2**, e132 (2004). 15138501 10.1371/journal.pbio.0020132 PMC406393

[47] J. Warringer , TRait variation in yeast is defined by population history. PLoS Genet. **7**, e1002111 (2011). 21698134 10.1371/journal.pgen.1002111 PMC3116910

[48] B. Papp, C. Pál, L. D. Hurst, Metabolic network analysis of the causes and evolution of enzyme dispensability in yeast. Nature **429**, 661–664 (2004). 15190353 10.1038/nature02636

[49] M. E. Hillenmeyer , The chemical genomic portrait of yeast: Uncovering a phenotype for all genes. Science **320**, 362–365 (2008). 18420932 10.1126/science.1150021 PMC2794835

[50] A. F. Kern , Divergent patterns of selection on metabolite levels and gene expression. BMC Ecol. Evol. **21**, 185 (2021). 34587900 10.1186/s12862-021-01915-5 PMC8482673

[51] B. R. Harrison, J. M. Hoffman, A. Samuelson, D. Raftery, D. E. L. Promislow, Modular evolution of the Drosophila metabolome. Mol. Biol. Evol. **39**, msab307 (2021), 10.1093/molbev/msab307. PMC8760934 34662414

[52] G. Liti , Population genomics of domestic and wild yeasts. Nature **458**, 337–341 (2009). 19212322 10.1038/nature07743 PMC2659681

[53] B. Szamecz , The genomic landscape of compensatory evolution. PLoS Biol. **12**, e1001935 (2014). 25157590 10.1371/journal.pbio.1001935 PMC4144845

[54] E. G. Bligh, W. J. Dyer, A rapid method of total lipid extraction and purification. Can. J. Biochem. Physiol. **37**, 911–917 (1959). 13671378 10.1139/o59-099

[55] W. Lu , Metabolomic analysis via reversed-phase ion-pairing liquid chromatography coupled to a stand alone orbitrap mass spectrometer. Anal. Chem. **82**, 3212–3221 (2010). 20349993 10.1021/ac902837x PMC2863137

[56] S. Reichling , Dynamic metabolome profiling uncovers potential TOR signaling genes. ELife **12**, e84295 (2023). 36598488 10.7554/eLife.84295 PMC9812406

[57] H. Link, T. Fuhrer, L. Gerosa, N. Zamboni, U. Sauer, Real-time metabolome profiling of the metabolic switch between starvation and growth. Nat. Methods **12**, 1091–1097 (2015). 26366986 10.1038/nmeth.3584

[58] A. Delabrière , proFIA: A data preprocessing workflow for flow injection analysis coupled to high-resolution mass spectrometry. Bioinformatics **33**, 3767–3775 (2017). 29036359 10.1093/bioinformatics/btx458

[59] T. Fuhrer, D. Heer, B. Begemann, N. Zamboni, High-throughput, accurate mass metabolome profiling of cellular extracts by flow injection-time-of-flight mass spectrometry. Anal. Chem. **83**, 7074–7080 (2011). 21830798 10.1021/ac201267k

[60] J. S. Shah , Distribution based nearest neighbor imputation for truncated high dimensional data with applications to pre-clinical and clinical metabolomics studies. BMC Bioinf. **18**, 114 (2017). 10.1186/s12859-017-1547-6 PMC5319174 28219348

[61] A. Mock , MetaboDiff: An R package for differential metabolomic analysis. Bioinformatics **34**, 3417–3418 (2018). 29718102 10.1093/bioinformatics/bty344 PMC6157071

[62] X.-X. Shen , Tempo and mode of genome evolution in the budding yeast subphylum. Cell **175**, 1533–1545.e20 (2018). 30415838 10.1016/j.cell.2018.10.023 PMC6291210

[63] V. Sarilar , Genome sequence of the type strain CLIB 1764T (=CBS 14374T) of the yeast species Kazachstania saulgeensis isolated from French organic sourdough. Genom. Data **13**, 41–43 (2017). 28725555 10.1016/j.gdata.2017.07.003 PMC5501885

[64] J. L. Gordon , Evolutionary erosion of yeast sex chromosomes by mating-type switching accidents. Proc. Natl. Acad. Sci. U.S.A. **108**, 20024–20029 (2011). 22123960 10.1073/pnas.1112808108 PMC3250169

[65] E. Baker , The genome sequence of Saccharomyces eubayanus and the domestication of lager-brewing yeasts. Mol. Biol. Evol. **32**, 2818–2831 (2015). 26269586 10.1093/molbev/msv168 PMC4651232

[66] L. G. Macías, M. Morard, C. Toft, E. Barrio, Comparative genomics between Saccharomyces kudriavzevii and S. cerevisiae applied to identify mechanisms involved in adaptation. Front. Genet. **10**, 187 (2019). 30930934 10.3389/fgene.2019.00187 PMC6425871

[67] D. R. Scannell , The awesome power of yeast evolutionary genetics: New genome sequences and strain resources for the Saccharomyces sensu stricto genus. G3 (Bethesda) **1**, 11–25 (2011). 22384314 10.1534/g3.111.000273 PMC3276118

[68] C. Holt, M. Yandell, MAKER2: An annotation pipeline and genome-database management tool for second-generation genome projects. BMC Bioinf. **12**, 491 (2011). 10.1186/1471-2105-12-491 PMC3280279 22192575

[69] P. Rice, I. Longden, A. Bleasby, EMBOSS: The european molecular biology open software suite. Trends Genet. **16**, 276–277 (2000). 10827456 10.1016/s0168-9525(00)02024-2

[70] D. M. Emms, S. Kelly, OrthoFinder: Phylogenetic orthology inference for comparative genomics. Genome Biol. **20**, 238 (2019). 31727128 10.1186/s13059-019-1832-y PMC6857279

[71] K. Katoh, D. M. Standley, MAFFT multiple sequence alignment software version 7: Improvements in performance and usability. Mol. Biol. Evol. **30**, 772–780 (2013). 23329690 10.1093/molbev/mst010 PMC3603318

[72] S. Capella-Gutiérrez, J. M. Silla-Martínez, T. Gabaldón, trimAl: A tool for automated alignment trimming in large-scale phylogenetic analyses. Bioinformatics **25**, 1972–1973 (2009). 19505945 10.1093/bioinformatics/btp348 PMC2712344

[73] A. Stamatakis, RAxML version 8: A tool for phylogenetic analysis and post-analysis of large phylogenies. Bioinformatics **30**, 1312–1313 (2014). 24451623 10.1093/bioinformatics/btu033 PMC3998144

[74] F.-J. Lapointe, G. Theodore Jr., A generalized permutation model for the analysis of cross-species data. J. Classif. **18**, 109–127 (2001).

[75] F. J. Rohlf, Comparative methods for the analysis of continuous variables: Geometric interpretations. Evolution **55**, 2143–2160 (2001). 11794776 10.1111/j.0014-3820.2001.tb00731.x

[76] E. Paradis, K., Schliep, ape 5.0: An environment for modern phylogenetics and evolutionary analyses in R. Bioinformatics **35**, 526–528 (2019). 30016406 10.1093/bioinformatics/bty633

[77] D. M. Bates, J. Pinheiro, Mixed-Effects Models in S and S-PLUS (Springer-Verlag, 2000), 10.1007/b98882 (28 July 2023).

[78] R. Tengölics, MTBLS9200: The metabolic domestication syndrome of budding yeast. Zenodo. https://zenodo.org/records/10680639. Deposited 19 February 2024. 10.1073/pnas.2313354121 PMC10945815 38457520

