# Supplementary material for: The metabolic domestication syndrome of budding yeast
Source: Proc Natl Acad Sci U S A. 2024 Mar 8;121(11):e2313354121. doi: 10.1073/pnas.2313354121 (PMC10945815; doi:10.1073/pnas.2313354121)
Supplement: Supplementary file 1 — Appendix 01 (PDF) [file pnas.2313354121.sapp.pdf]

PNAS

Supporting Information for

The metabolic domestication syndrome of budding yeast

Roland Tengölics*, Balázs Szappanos*, Michael Mülleder, Dorottya Kalapis, Gábor Grézal, Csilla Sajben, Federica Agostini, João Benhur Mokochinski, Balázs Bálint, László G. Nagy, Markus Ralser, Balázs Papp

Correspondence to: markus.ralser@charite.de (M.R.), balazs.papp@brc.hu (B.P.)
*These authors contributed equally to this work

This PDF file includes:

Supporting text
Figures S1 to S13
Tables S1 to S5
Legends for Datasets S1 to S12

Other supporting materials for this manuscript include the following:

Datasets S1 to S12

Supporting Information text

Targeted quantification of TCA cycle intermediates and pyruvic acid

We followed an established method (1) with modification for the quantification of TCA cycle intermediates and pyruvate. We cultivated 1100 µl culture/sample as for amino acid quantification. For optical density determination, 100 µl culture was used, and 1000 µl were harvested by centrifugation at 4 °C for 3 minutes. In one experimental batch 4 replicates were cultivated and measured. Pellets were extracted as described in non-targeted metabolomics, except for the second extraction only 150 µl extraction solution was used. Cleared extracts were unified and dried in vacuum. Died extracts were stored at -80 until LC-MS measurement in 'V' bottom polypropylene plates. Dried extracts were dissolved in 150 µl LC-MS grade water with shaking for 10 minutes at room temperature at 900 rpm using Titramax 101. Redisolved extracts were cleared with centrifugation at 4C for 5 minutes at 4500 rpm. They were placed into 384-well polystyrene plates and covered with a seal. Pooled extracts (quality control samples, QCs) were prepared by joining the dissolved extracts of samples for each experimental batch separately. During analysis, a quality control sample (QC) was assessed 20 times/experimental batch. It was used to (i) monitor analytical performance and calculate the technical variation in each metabolite and (ii) perform QC-RLSC normalization. The calibration curve was injected 4 times / experimental batch. Samples were excluded from the analysis if the general response to organic acids was too low.

Organic acids were separated using a Waters HSS T3 column (1.7 µm, 2.1 mm X 100 mm) on a liquid chromatography (Waters ACQUITY Premier) and tandem mass spectrometry (Waters TQS-Micro) system. Buffer A was composed of LC-MS grade water (VWR - 83645.320), 0.175% formic acid (Sigma 5.33002) and buffer B of 100 acetonitrile (VWR - 83640.320), The gradient elution was performed at a constant flow rate of 0.25 ml/min. Starting conditions were 100% A until 2.3 minutes. From 2.3 min to 3 min, B was increased to 30% with a gradient profile of 10, and kept for 0.5 min before returning to initial conditions. The column was then equilibrated for another 1.2 minutes, resulting in a 4.7-minute chromatographic run. Compounds were identified by matching retention time and fragmentation with commercially available standards (Table S5, Sigma-Aldrich). For MS/MS acquisition - 0.5kV capillary voltage, desolvation gas temperature was 600 °C and desolvation gas flow was 1000 l/min. Signals for free amino acids were then acquired in MRM mode in Masslynx software. Quantification was performed using external calibration with the ratio of internal standard's peak area and target compound's quantification ion (2). Intensities were normalized as in amino acid quantification, i.e. using PQN normalization and linear regression between optical density at the time of sampling and metabolite intensities (3).

Genome annotation

To obtain consistent genome annotation across the studied yeast isolates, we annotated all the 71 budding yeast genomes using the MAKER genome annotation pipeline v2.31.10 (4). Genome annotation using MAKER occurs in an iterative manner and relies on multiple inputs, some of which were universal to all genomes (e.g., homology evidence), whereas the others were species-specific (e.g., parameters for ab initio gene predictors). The procedure described below was followed for the annotation of all 71 genomes. The homology evidence used in our genome annotation consists of fungal protein sequences in the SwissProt database (5) (release 2020_04; ftp://ftp.ebi.ac.uk/pub/databases/uniprot/current_release/knowledgebase/taxonomic_divisions/uniprot_sprot_fungi.dat.gz). Three ab initio gene predictors were used with the MAKER pipeline, including SNAP v2017-03-01 (6), and AUGUSTUS v 3.3.2 (7). SNAP was trained for each individual genome, while the pretrained ‘‘Saccharomyces’’ model was used for AUGUSTUS prediction. For soft-masking the genome sequences RepeatMasker v4.0.7 (8)(http://www.repeatmasker.org) with the library Repbase library release-20170127 and the ‘‘-species’’ parameter set to ‘‘Saccharomyces’’ for all genomes were used. The genome annotation pipeline was designed based on the MAKER genome annotation pipeline (4) (https://reslp.github.io/blog/My-MAKER-Pipeline/). These steps are the following: 1; Run MAKER with EST and/or protein evidence. 2; Train SNAP based on results from Step 1. 3; Run MAKER with the training results from Step 2. 4; Train SNAP a second time with results from Step 3. 5; Run MAKER with the training results from Step 4. 6; Train AUGUSTUS. 7; Run again like 5 but include AUGUSTUS training file and set keep_preds=1. 8; Run MAKER with SNAP, AUGUSTUS. 7 out of the 71 published genomes (~10%) analyzed in our study have previously been annotated. To evaluate the quality of our genome annotations, we performed direct comparisons between our annotations and existing ones for the same species. Genome annotations of 7 strains (Ksau_CBS14374T, Sarb_CBS10644, Scas_CBS4309_v1, Sjur_D5088, Spar_N44, Spar_UFRJ50816, Spar_YPS138) were downloaded from their respective sources and compared to the corresponding annotations generated in our study using Cuffcompare v2.2.1 (9). Moreover, *Saccharomyces cerevisiae* S88C strain annotation was used as a highly curated reference (Downloaded from ftp://ftp.ensembl.org/pub/release-100/fasta/saccharomyces_cerevisiae/dna/). Using the existing annotations as the references, our predictions achieved high levels of specificity (ranging from 96.8% to 98.8%, with an average of 98.1%) and sensitivity (ranging from 95.0% to 99.3%, with an average of 98.5%) at the base pair level, as well as contained few missing exons (the fraction of exons missing ranged from 0.6% to 35.7%, with an average of 9.5%) and genes (the fraction of genes missing ranged from 0.2% to 6.8%, with an average of 4.6%). Note that all of these calculations assume that the previously published annotations contain no errors, which might inflate

estimates of inaccuracies in our annotations. To further assess the quality of genome annotations, we used BUSCO 4.0.5 (10) to calculate annotation completeness, based on the presence of single-copy orthologs. The fraction of these orthologs ranged from 96.1% to 99.4%, with an average of 98.9%). Re-annotation of the S288C reference strain with our pipeline resulted in 99.4% overlap with the total 2137 BUSCO Saccharomycetes group v1.0 genes.

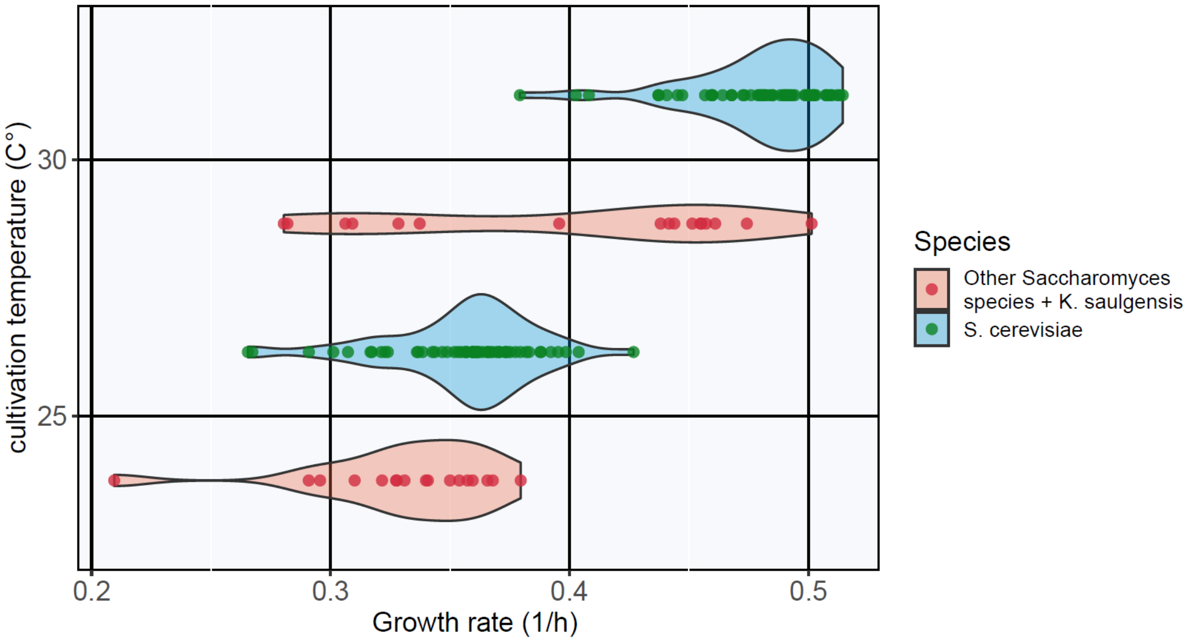

Fig. S1. Effect of temperature on growth rates of *S. cerevisiae* and other yeast species. We measured growth rates of all genotypes at two temperatures: 25°C and 30°C. The cultivation temperature affects the growth rate distributions both within *S. cerevisiae* and across all yeast species. A higher growth rate heterogeneity was observed for non-cerevisiae isolates at 30°C compared to 25°C (median absolute deviations: 0.0357 at 30°C versus 0.0252 at 25°C). Importantly, the average growth rates of *S. cerevisiae* and non-cerevisiae isolates do not differ significantly at 25°C (P = 0.062, two-sided t-test), which makes metabolome measurements at 25°C better suited for interspecies comparisons.

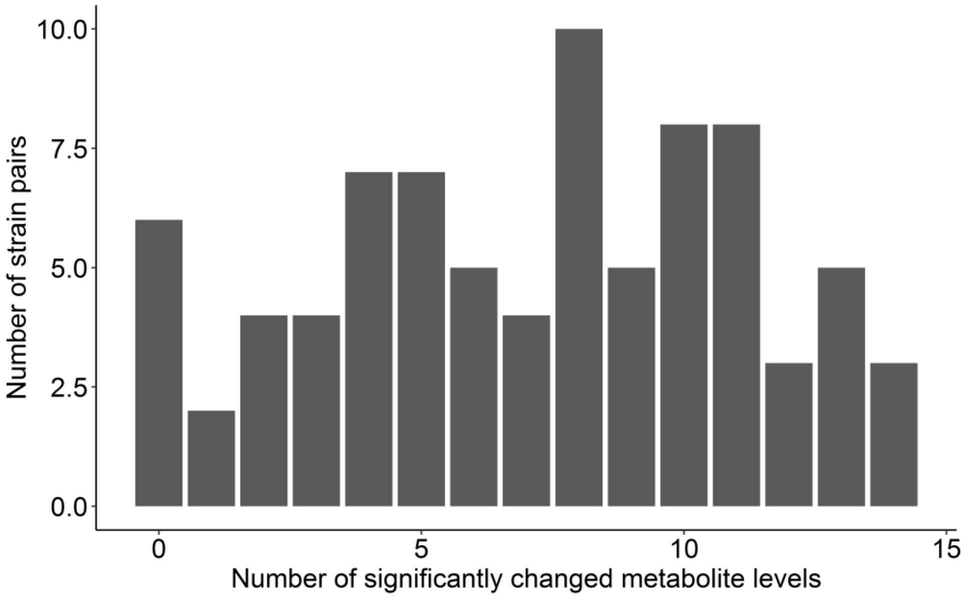

Fig. S2. Distribution of the number of significantly changed amino acid levels for *S. cerevisiae* strain pairs with identical metabolic networks. For each strain pairs T-test was used to calculate difference between the replicates of the two strains. FDR method was used to adjust the p values for multiple testing. FDR values under 0.1 were considered significant.

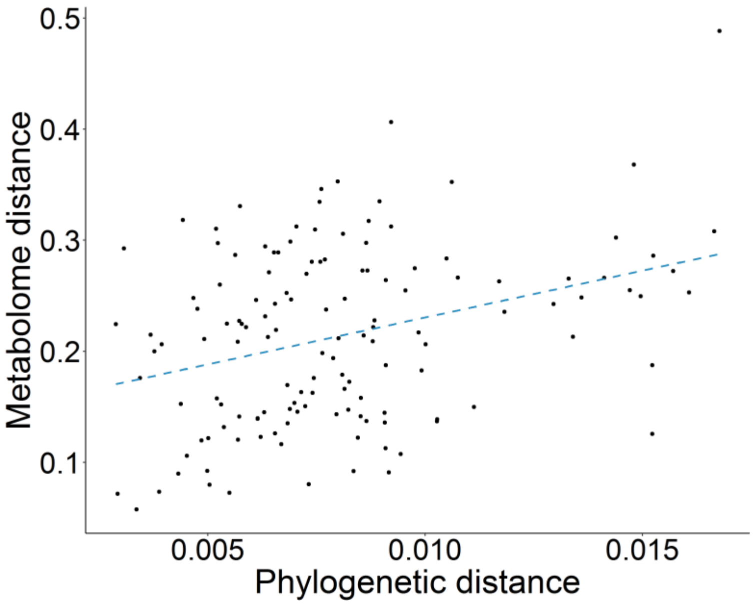

Fig. S3. Lack of significant correlation between phylogenetic distance and amino acid metabolome divergence across pairs of *S. cerevisiae* populations (r = 0.33, p = 0.33, phylogenetic Mantel test; sample size = 136 pairs). The dashed (blue) line shows the linear regression line.

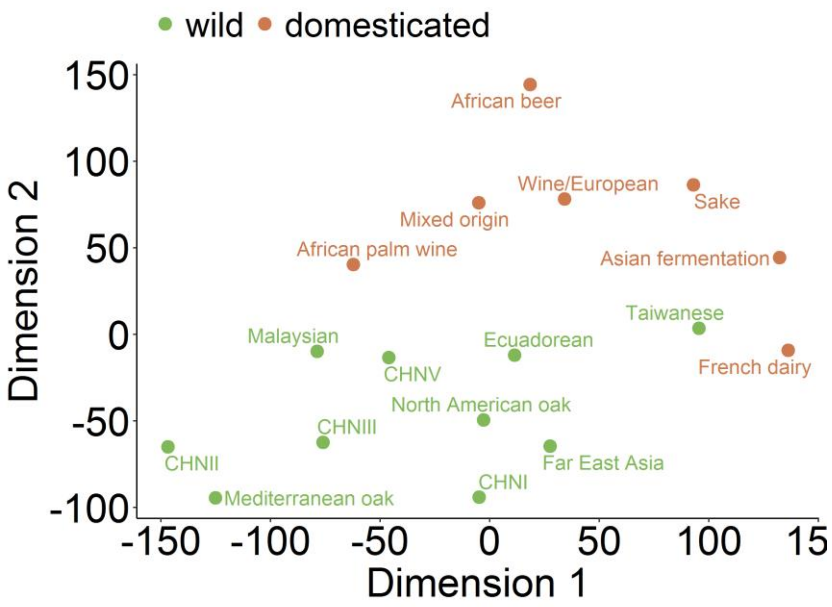

Fig. S4. Separation between wild and domesticated *S. cerevisiae* populations in the non-targeted metabolome. t-SNE algorithm was applied on the data. Green and orange dots indicate wild and domesticated populations, respectively.

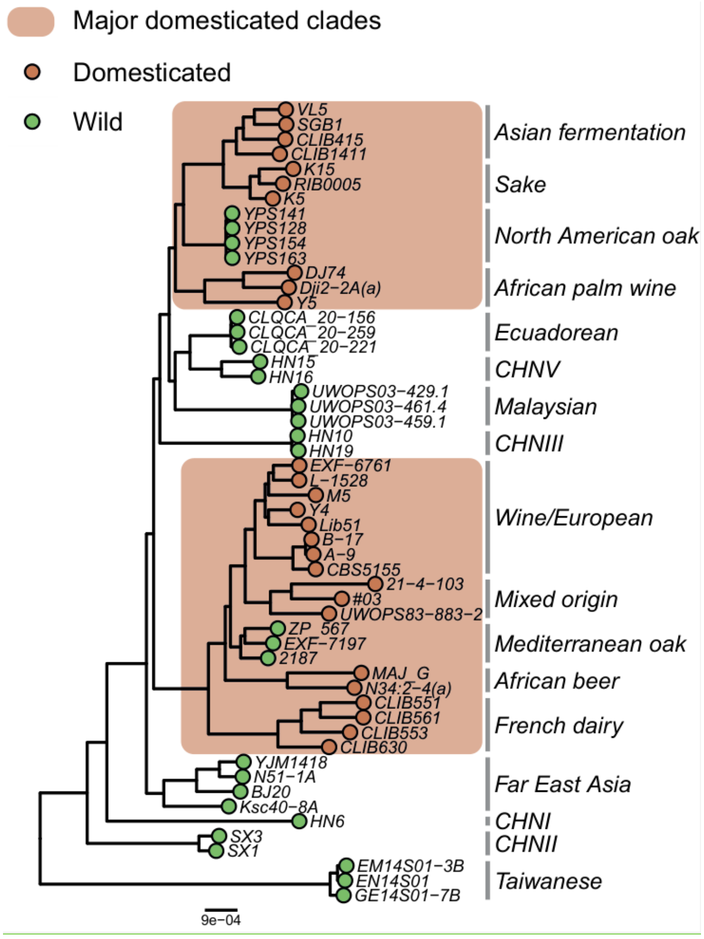

Fig. S5. Phylogenetic tree of *S. cerevisiae* strains highlighting domesticated clades. Boxes with shading highlight the two widely separated clades predominantly containing domesticated populations.

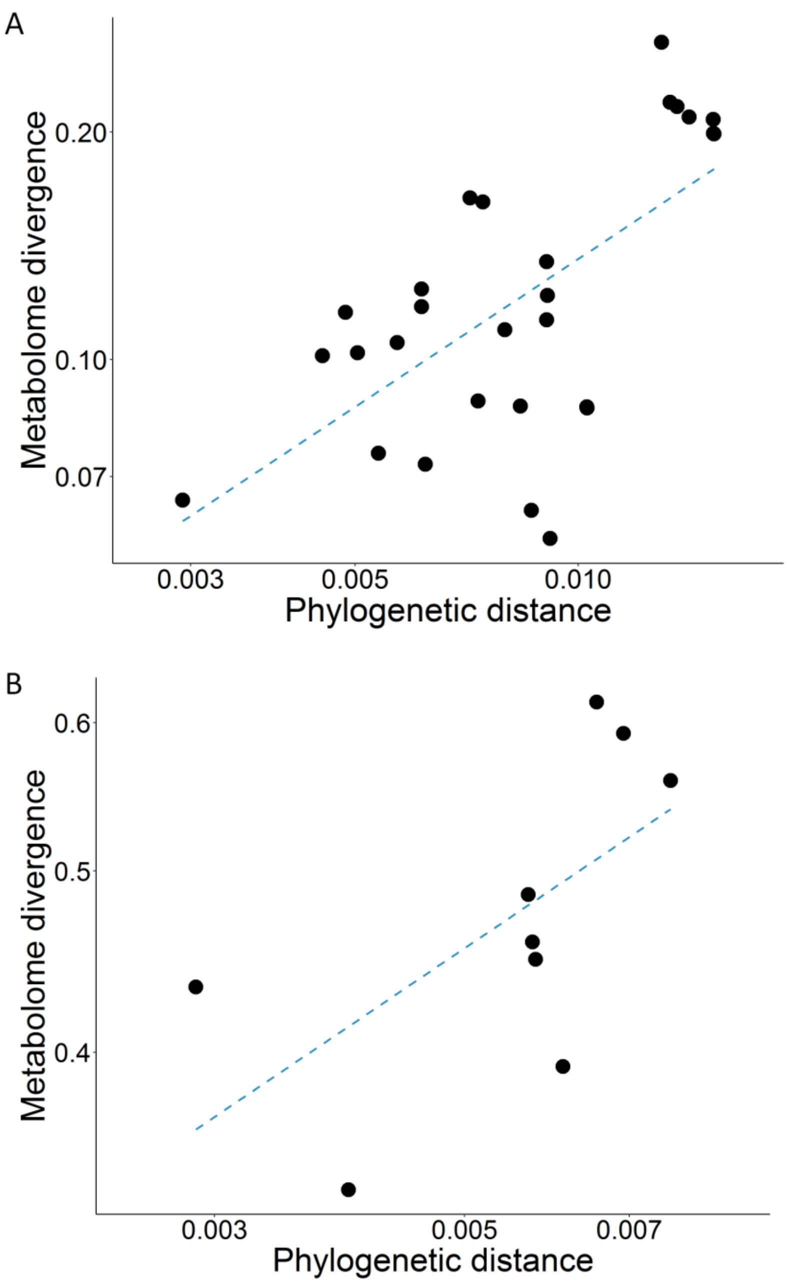

Fig. S6. Metabolome divergence of non-amino acid metabolites as a function of phylogenetic distance among *S. cerevisiae* population pairs. Note that these metabolites were measures by non-targeted metabolomics and therefore are considered as putative metabolites. A) Pairs of wild *S. cerevisiae* populations (N = 28). North American oak and Mediterranean oak populations are excluded, as their closest relatives are domesticated populations. Dashed line (blue) indicates the linear regression line. B) Pairs of domesticated populations that come from the same domesticated clades (N = 9). Dashed line (blue) indicates the linear regression line.

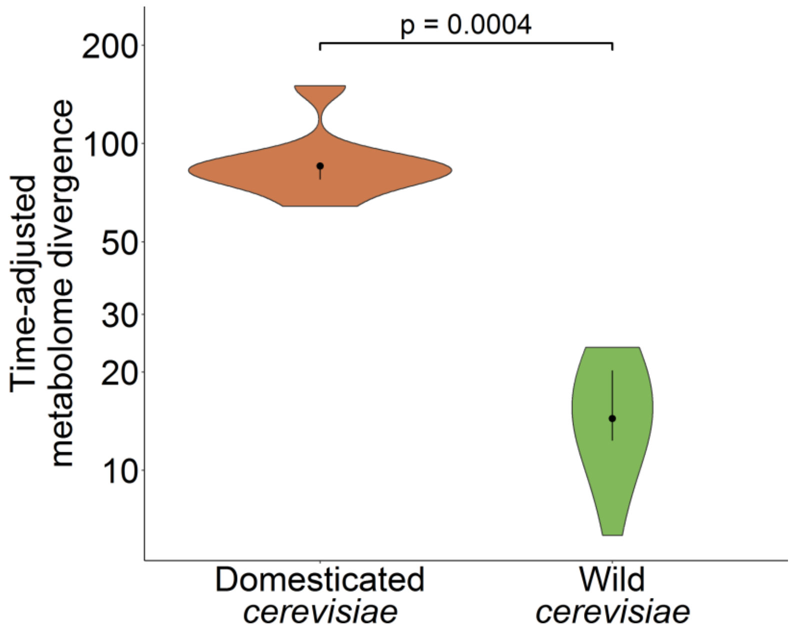

Fig. S7. Comparison of time-adjusted non-amino acid metabolome divergence among pairs of domesticated (N = 9) and wild (N = 28) *S. cerevisiae* populations. Note that these metabolites were measures by non-targeted metabolomics and therefore are considered as putative metabolites. P-value was determined using a permutation test (see Methods).

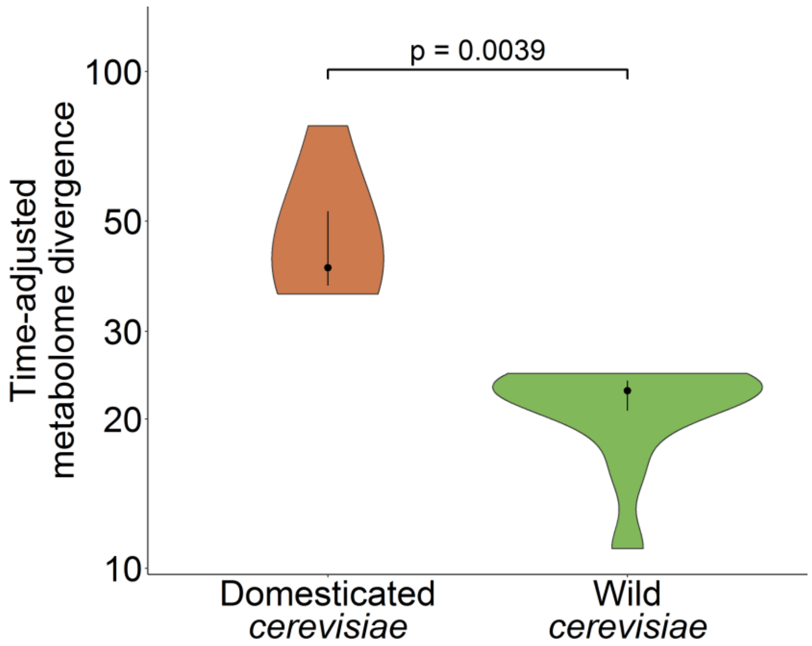

Fig. S8. Comparison of time-adjusted amino acid metabolome divergence among pairs of domesticated (N = 9) and wild (N = 12) *S. cerevisiae* populations. Note that Wild population pairs that are phylogenetically more distant than the most distantly related domesticated population pairs were excluded from the analysis. P-value was determined using a permutation test.

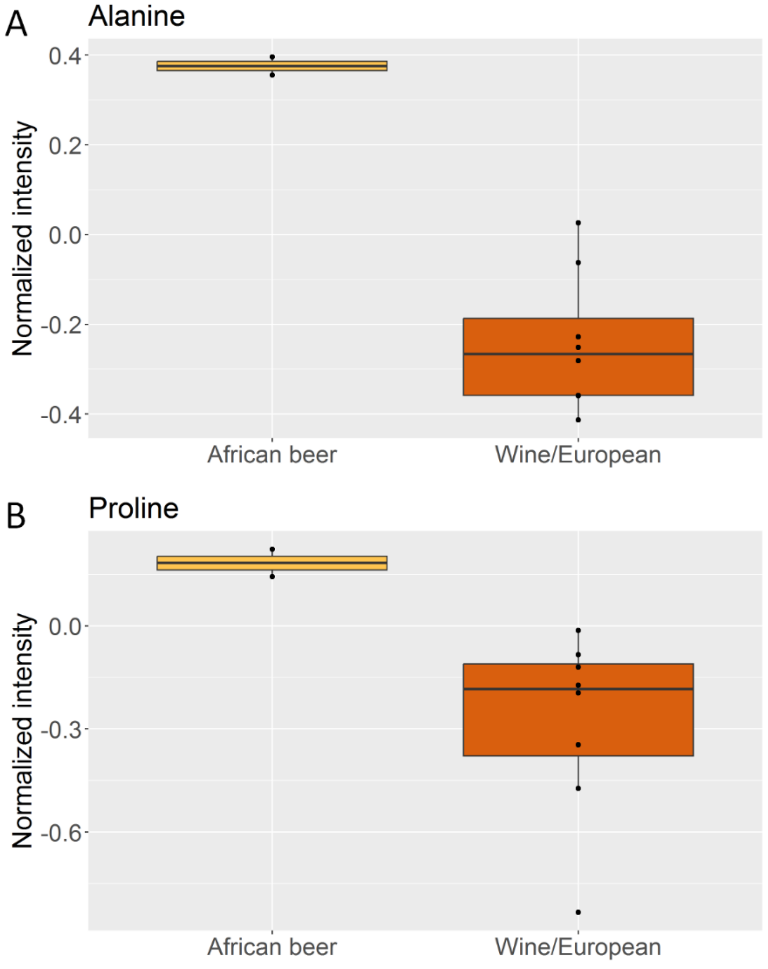

Fig. S9. Alanine (A) and proline (B) levels in the African beer and Wine populations. Dots represent individual strains (N=2 for African beer and N=7 for Wine/European). Differences are significant after false discovery rate correction (two-sided t-tests: FDR-adjusted p=0.0001 and p=0.043 for alanine and proline, respectively, see also Dataset S10).

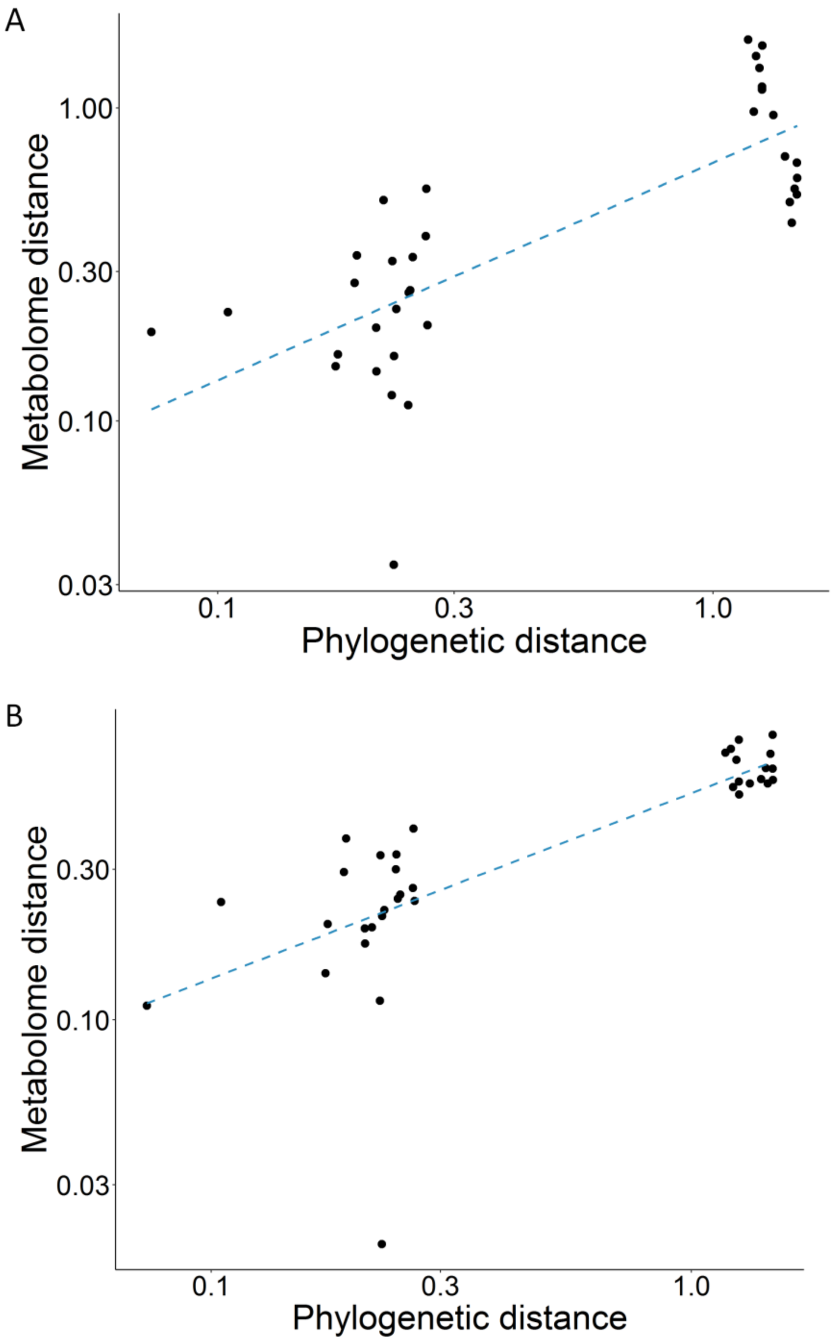

Fig. S10. Correlation between metabolome and phylogenetic distance among yeast species pairs (N = 36). A) Amino acid metabolome distance as a function of phylogenetic distance (r=0.71 and p=0.011, phylogenetic Mantel test). Dashed line (blue) indicates the linear regression line. B) Non-amino acid metabolome distance as a function of phylogenetic distance (r=0.91 and p=4.6e-4, phylogenetic Mantel test). Dashed line (blue) indicates the linear regression line. Note that these metabolites were measures by non-targeted metabolomics and therefore are considered as putative metabolites.

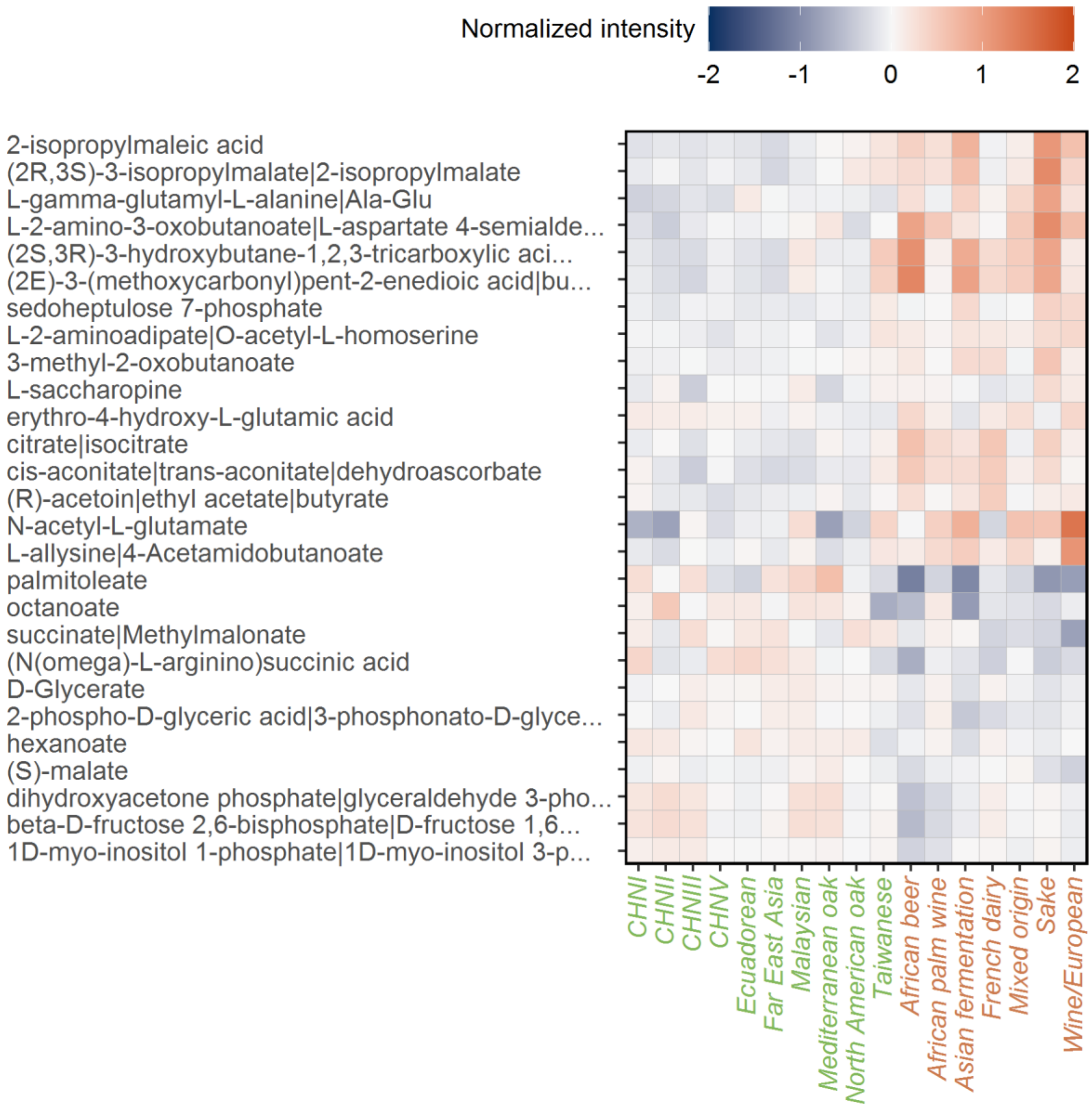

Fig. S11. Population-average levels of non-amino acid metabolites showing domestication signature in wild and domesticated *S. cerevisiae* populations. Note that these metabolites were measures by non-targeted metabolomics and therefore are considered as putative metabolites. Wild and domesticated populations are marked as green and orange, respectively. Metabolite levels are studentized for each metabolite for visualization purposes.

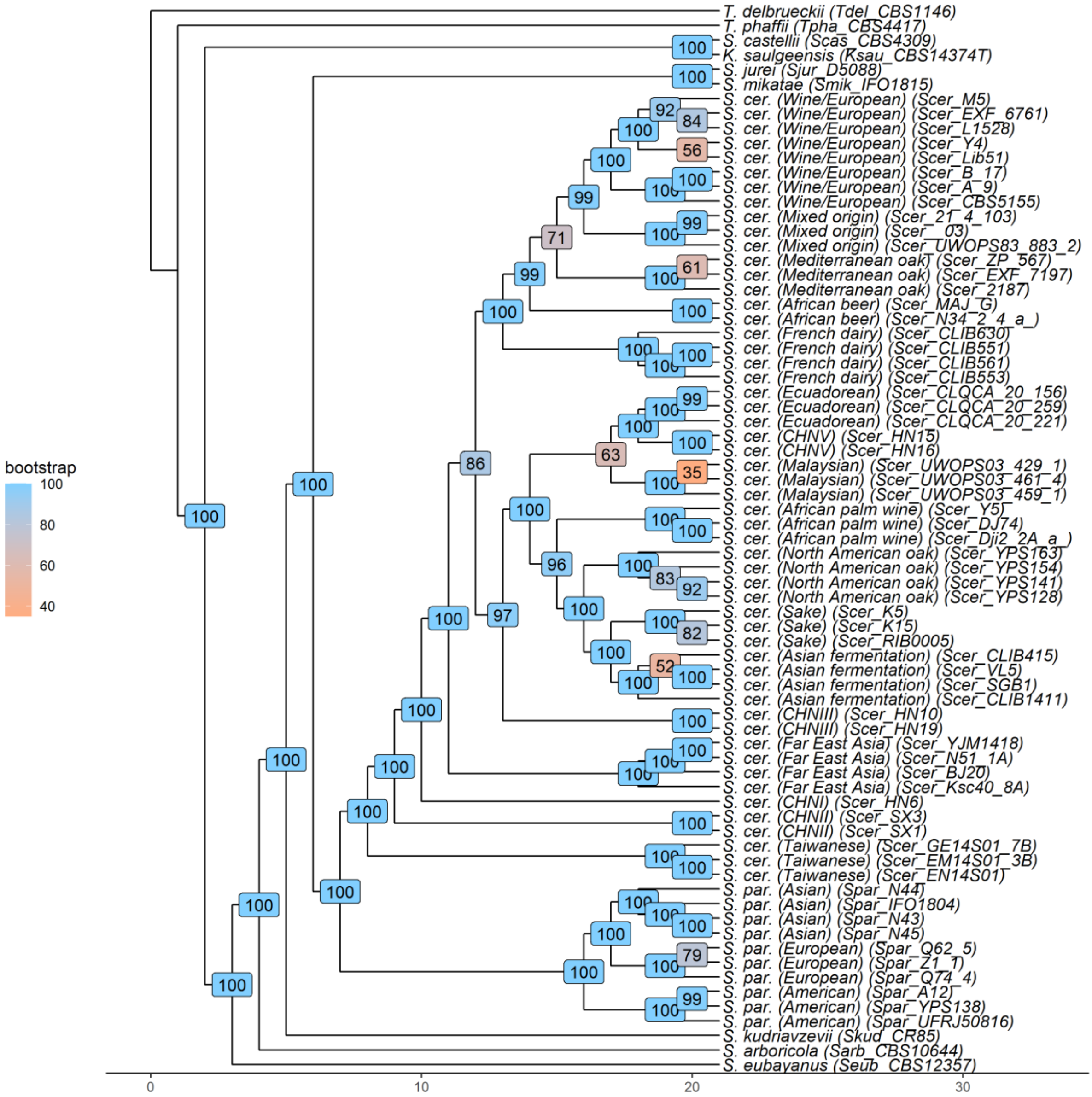

Fig. S12. Bootstrap support of internal nodes on the phylogenetic tree of studied yeast strains.

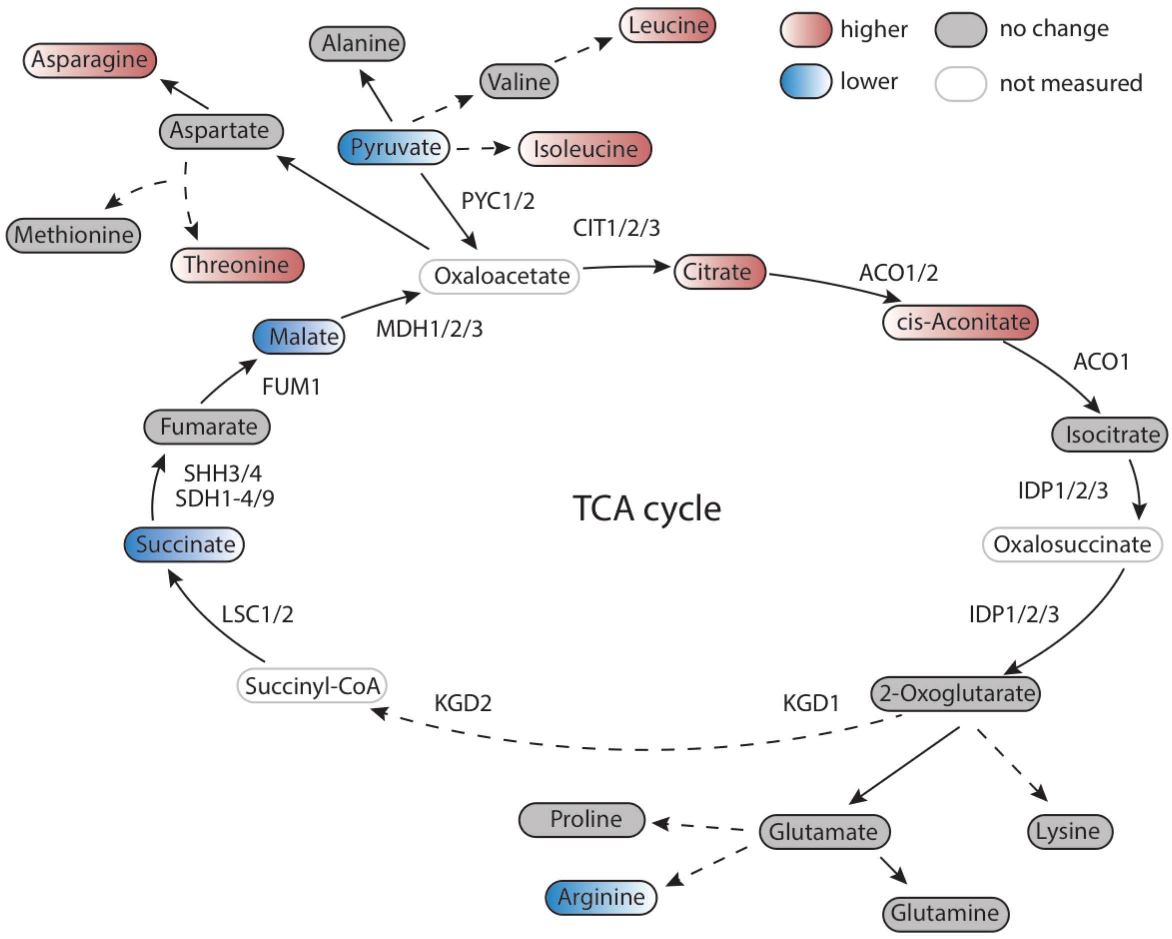

Fig. S13. Schematic of the TCA cycle and amino acid biosynthesis. Metabolites with increased or decreased levels in domesticated populations are marked as red or blue, respectively. Metabolites showing no domestication signature and unmeasured metabolites are denoted with filled gray and white labels, respectively.

Table S1. ANOVA testing of population differences in metabolite levels for each amino acid. White-adjusted ANOVA test was applied to control for heteroscedasticity. Populations with less than 2 strains were excluded from the analysis. P values were adjusted for multiple testing with the FDR method. ‘All_populations_FDR’ and ‘cerevisiae_populations_FDR’ columns show the FDR values for the tests on all studied 27 populations and the 17 *S. cerevisiae* populations, respectively. Significance cutoff for FDR values was 0.1.

**Metabolite**	**All populations FDR**	***S. cerevisiae* populations FDR**
Alanine	4.49E-18	1.85E-11
Arginine	1.35E-06	1.87E-05
Asparagine	2.43E-15	1.30E-13
Aspartic acid	2.65E-11	4.42E-10
Glutamic acid	4.28E-11	4.42E-10
Glutamine	2.04E-03	4.53E-03
Glycine	2.70E-16	3.37E-14
Histidine	1.19E-08	1.18E-07
Isoleucine	2.26E-04	2.28E-04
Leucine	4.82E-10	1.82E-08
Lysine	1.28E-08	1.47E-03
Methionine	5.94E-08	1.68E-04
Phenylalanine	1.24E-16	4.42E-10
Proline	3.44E-06	3.22E-05
Serine	1.81E-14	2.79E-13
Threonine	4.55E-04	8.82E-04
Tryptophan	6.41E-05	7.45E-04
Tyrosine	3.46E-01	4.28E-01
Valine	7.05E-04	8.05E-01

Table S2. Yield distances between species.

**Species 1**	**Species 2**	**Yield distance**
Saccharomyces arboricola	*Saccharomyces castelli*	0.007661891
Saccharomyces castelli	*Saccharomyces cerevisiae*	0.007661891
Saccharomyces arboricola	*Saccharomyces cerevisiae*	0
Saccharomyces castelli	*Saccharomyces eubayanus*	0.000383118
Saccharomyces arboricola	*Saccharomyces eubayanus*	0.007333648
Saccharomyces cerevisiae	*Saccharomyces eubayanus*	0.007333648
Saccharomyces castelli	*Saccharomyces kudriavzevii*	0.000383118
Saccharomyces arboricola	*Saccharomyces kudriavzevii*	0.007333648
Saccharomyces cerevisiae	*Saccharomyces kudriavzevii*	0.007333648
Saccharomyces eubayanus	*Saccharomyces kudriavzevii*	0

Saccharomyces castelli	*Saccharomyces mikatae*	0.000383118
Saccharomyces arboricola	*Saccharomyces mikatae*	0.007333648
Saccharomyces cerevisiae	*Saccharomyces mikatae*	0.007333648
Saccharomyces eubayanus	*Saccharomyces mikatae*	0
Saccharomyces kudriavzevii	*Saccharomyces mikatae*	0
Saccharomyces castelli	*Saccharomyces paradoxus*	0.007674382
Saccharomyces arboricola	*Saccharomyces paradoxus*	9.45777E-05
Saccharomyces cerevisiae	*Saccharomyces paradoxus*	9.45777E-05
Saccharomyces eubayanus	*Saccharomyces paradoxus*	0.00723907
Saccharomyces kudriavzevii	*Saccharomyces paradoxus*	0.00723907
Saccharomyces mikatae	*Saccharomyces paradoxus*	0.00723907

Table S3. Climate information (tropical or continental) and geographic location information on the continent level for each *S. cerevisiae* population.

**Population**	**Climate**	**Continent**
African beer	Tropical	Africa
African palm wine	Tropical	Africa
Asian fermentation	Tropical	Asia
CHNI	Tropical	Asia
CHNII	Continental	Asia
CHNIII	Tropical	Asia
CHNV	Tropical	Asia
Ecuadorean	Tropical	America
Far East Asia	Continental	Asia
French dairy	Continental	Europe
Malaysian	Tropical	Asia
Mediterranean oak	Continental	Europe
Mixed origin	Continental	Europe
North American oak	Continental	America
Sake	Continental	Asia
Taiwanese	Tropical	Asia
Wine/European	Continental	Europe

Table S4. Results of phylogenetic ANOVA tests (see Methods) comparing amino acid metabolite levels across different climate or geographic location.

**Metabolite**	**Climate p**	**Climate FDR**	**Continent p**	**Continent FDR**
Alanine	0.9720	0.9720	0.0825	0.5222
Arginine	0.7370	0.8531	0.2801	0.6785
Asparagine	0.6481	0.8531	0.3571	0.6785
Aspartic acid	0.8082	0.8531	0.3430	0.6785

Glutamic acid	0.6169	0.8531	0.0296	0.5222
Glutamine	0.6766	0.8531	0.1900	0.6785
Glycine	0.7974	0.8531	0.3256	0.6785
Histidine	0.6082	0.8531	0.2735	0.6785
Isoleucine	0.4964	0.8531	0.1712	0.6785
Leucine	0.4517	0.8531	0.9807	0.9807
Lysine	0.1338	0.8460	0.8468	0.9465
Methionine	0.5591	0.8531	0.7371	0.9193
Phenylalanine	0.7382	0.8531	0.7741	0.9193
Proline	0.0424	0.8054	0.0551	0.5222
Serine	0.2050	0.8460	0.9383	0.9807
Threonine	0.7596	0.8531	0.4270	0.7375
Tryptophan	0.2226	0.8460	0.6258	0.8593
Tyrosine	0.7196	0.8531	0.6331	0.8593
Valine	0.1564	0.8460	0.5798	0.8593

Table S5. Retention time and transition for standards of TCA cycle metabolites.

Metabolite name	Retention time (min)	Transition	Manufacturer	Cat number
Succinic acid	3.29	117.2 > 73.05	Afha Aesar	33272
Cis-aconitic acid	2.96	173 > 85	Sigma-Aldrich	A3412-1G
Alpha-ketoglutaric acid	1.95	145 > 57.15	Reanal	NA
Malic acid	1.57	133.2 > 114.91	Sigma-Aldrich	02288-50G
Fumaric acid	2.95	115.2 > 27.5	Sigma-Aldrich	47910-5G
Isocitric acid	1.59	191.1 > 111.05	Sigma-Aldrich	I-1252
Pyruvic acid	1.57	87 > 42.8	Sigma-Aldrich	P2256-100G
Citric acid	2.54	191.1 > 111.1	Thermo	22869.36
Fumaric acid-2,3-d_2	2.95	118 > 30	Sigma-Aldrich	486671-5G
SUCCINIC-2,3-13C2	3.29	120 > 75	Sigma-Aldrich	488364-100MG

Dataset S1. Amino acid levels in yeast strains.

Dataset S2. Intensities of non-amino acid metabolites in yeast strains. Note that these metabolites were measures by non-targeted metabolomics and therefore are considered as putative metabolites.

Dataset S3. Levels of TCA cycle metabolites in yeast strains.

Dataset S4. Phylogenetic tree of yeast strains. Strain ids of the tree match the strain ids of Dataset S1 ('Strain-level data' sheet, 'Unique strain id used in the study' column).

Dataset S5. Phylogenetic tree of yeast populations. Population names on the tree are derived from species and population names of Fig. 1/A.

Dataset S6. List of yeast strains used in our study.

Dataset S7. ANOVA testing of population differences in non-targeted metabolic feature levels. White-adjusted ANOVA test was applied to control for heteroscedasticity. Populations with less than 2 strains were excluded from the analysis. P values were adjusted for multiple testing with the FDR method. Significance cutoff for FDR values was 0.1.

Dataset S8. Yield distances between *S. cerevisiae* populations.

Dataset S9. Number of significant amino acid level differences for *S. cerevisiae* strain pairs with identical theoretical amino acid yields. Yields were considered identical if yield distance was smaller than 1e-3. For each strain pairs T-test was used to calculate difference between the replicates of the two strains. FDR method was used to adjust the p values for multiple testing. FDR values under 0.1 were considered significant.

Dataset S10. Differences between amino acid levels between domesticated *S. cerevisiae* populations. For each population pair t-test was used to determine whether the strains of the two populations have different amino acid level or not. FDR method was applied for each amino acid to control for multiple testing.

Dataset S11. Domestication associated amino acids, TCA cycle metabolites, and non-targeted metabolome traits.

Dataset S12. Comparison of amino acid and non-amino acid metabolome between the two monophyletic domesticated clades.

SI references

1. O. Al Kadhi, A. Melchini, R. Mithen, S. Saha, Development of a LC-MS/MS Method for the Simultaneous Detection of Tricarboxylic Acid Cycle Intermediates in a Range of Biological Matrices. *J Anal Methods Chem* **2017**, 5391832 (2017).

2. R. Rathod, B. Gajera, K. Nazir, J. Wallenius, V. Velagapudi, Simultaneous Measurement of Tricarboxylic Acid Cycle Intermediates in Different Biological Matrices Using Liquid Chromatography–Tandem Mass Spectrometry; Quantitation and Comparison of TCA Cycle

Intermediates in Human Serum, Plasma, Kasumi-1 Cell and Murine Liver Tissue. *Metabolites* **10**, 103 (2020).

3. M. Zampieri, *et al.*, High-throughput metabolomic analysis predicts mode of action of uncharacterized antimicrobial compounds. *Science Translational Medicine* **10**, eaal3973 (2018).

4. C. Holt, M. Yandell, MAKER2: an annotation pipeline and genome-database management tool for second-generation genome projects. *BMC Bioinformatics* **12**, 491 (2011).

5. The UniProt Consortium, UniProt: the Universal Protein Knowledgebase in 2023. *Nucleic Acids Research* **51**, D523–D531 (2023).

6. I. Korf, Gene finding in novel genomes. *BMC Bioinformatics* **5**, 59 (2004).

7. M. Stanke, R. Steinkamp, S. Waack, B. Morgenstern, AUGUSTUS: a web server for gene finding in eukaryotes. *Nucleic Acids Research* **32**, W309–W312 (2004).

8. S. Tempel, “Using and Understanding RepeatMasker” in *Mobile Genetic Elements: Protocols and Genomic Applications*, Methods in Molecular Biology., Y. Bigot, Ed. (Humana Press, 2012), pp. 29–51.

9. C. Trapnell, *et al.*, Transcript assembly and quantification by RNA-Seq reveals unannotated transcripts and isoform switching during cell differentiation. *Nat Biotechnol* **28**, 511–515 (2010).

10. M. Manni, M. R. Berkeley, M. Seppey, E. M. Zdobnov, BUSCO: Assessing Genomic Data Quality and Beyond. *Current Protocols* **1**, e323 (2021).
